# Oat Peptides Alleviate Dextran Sulfate Sodium Salt-Induced Colitis by Maintaining the Intestinal Barrier and Modulating the Keap1-Nrf2 Axis

**DOI:** 10.3390/nu15245055

**Published:** 2023-12-09

**Authors:** Zhong-Hao Ji, Wen-Yin Xie, Pei-Sen Zhao, Hong-Yu Wu, Wen-Zhi Ren, Jin-Ping Hu, Wei Gao, Bao Yuan

**Affiliations:** 1 Department of Laboratory Animals, College of Animal Sciences, Jilin University, Changchun 130062, China; jizh21@mails.jlu.edu.cn (Z.-H.J.); xiewy9918@mails.jlu.edu.cn (W.-Y.X.); zhaops22@mails.jlu.edu.cn (P.-S.Z.); wuhy21@mails.jlu.edu.cn (H.-Y.W.); renwz@jlu.edu.cn (W.-Z.R.); hujp@jlu.edu.cn (J.-P.H.); 2 Department of Basic Medicine, Changzhi Medical College, Changzhi 046000, China; 3 Jilin Academy of Agricultural Sciences, Jilin 132101, China; 4 Changchun National Experimental Animal Center, Jilin University, Changchun 130062, China

**Keywords:** IBD, oats, peptides, Keap1-Nrf2, gut bacteria

## Abstract

The prevalence of inflammatory bowel disease (IBD) is progressively rising each year, emphasizing the significance of implementing rational dietary interventions for disease prevention. Oats, being a staple agricultural product, are abundant in protein content. This study aimed to investigate the protective effects and underlying mechanisms of oat peptides (OPs) in a mouse model of acute colitis induced by dextran sulfate sodium salt (DSS) and a Caco-2 cell model. The findings demonstrated that intervention with OPs effectively mitigated the symptoms associated with DSS-induced colitis. The physicochemical characterization analysis demonstrated that the molecular weight of the OPs was predominantly below 5 kDa, with a predominant composition of 266 peptides. This study provides further evidence of the regulatory impact of OPs on the Keap1-Nrf2 signaling axis and elucidates the potential role of WGVGVRAERDA as the primary bioactive peptide responsible for the functional effects of OPs. Ultimately, the results of this investigation demonstrate that OPs effectively mitigate DSS-induced colitis by preserving the integrity of the intestinal barrier and modulating the Keap1-Nrf2 axis. Consequently, these findings establish a theoretical foundation for the utilization of OPs as dietary supplements to prevent the onset of IBD.

## 1. Introduction

Inflammatory bowel disease (IBD), which includes ulcerative colitis (UC) and Crohn’s disease (CD), is a chronic inflammatory disease. The incidence of IBD is increasing annually, and the disease imposes a very large burden on society [1]. The pathogenesis of IBD is unclear, but it is currently believed that genetics, the environment, the intestinal barrier, and the immune response are the main factors influencing its development [2]. Currently, the main treatment methods for IBD include glucocorticoids, immunomodulators (such as azathioprine), and biologics. The 2023 edition of the Chinese Ulcerative Colitis Diagnosis and Treatment Guidelines indicates that 5-aminosalicylic acid (5-ASA) is the preferred medication for inducing remission in mild-to-moderate active UC. In cases where 5-ASA is ineffective, corticosteroids and biologics are considered for inducing remission. Among them, tofacitinib and upadacitinib are oral small-molecule Janus kinase (JAK) inhibitors that have been approved in some countries for treating moderate-to-severe active UC [3,4]. Ustekinumab is an IL-12 and IL-23 inhibitor, and the clinical trial results suggest its ability to alleviate clinical symptoms in CD patients [5]. A large multicenter prospective cohort study demonstrated favorable outcomes with anti-tumor necrosis factor drugs infliximab and adalimumab in the treatment of pediatric CD [6]. However, these drugs have limited therapeutic effects and can have serious side effects when taken for long durations. Therefore, it is important to develop a low-cost, highly active, and safe dietary supplement for the prevention of IBD.

Oats (*Avena sativa*) are important agricultural products that are rich in nutritional value and have several benefits for human health [7]. For example, oats have antioxidant activities [8,9], which may be related to the phenolic substances present [10,11,12,13]. The β-glucan in oats has antifungal effects [14] and can alleviate dextran sulfate sodium salt (DSS)-induced enteritis [15], an effect that may be related to its modulatory effect on intestinal microbial metabolism [16]. In addition, the soluble fiber in oats can alleviate trinitrobenzene sulfonic acid (TNBS)-induced enteritis by inducing regulatory T-cell (Treg) differentiation [17].

Oats are rich in protein (12–20%) [18,19], which makes them an important source of bioactive peptides. Oat-derived peptides have antimicrobial [20] and metal-chelating [21] effects. In addition, recent studies have shown that oat-derived antimicrobial peptides alleviate DSS-induced colitis and have regulatory effects on intestinal microbial composition and metabolism [20]. However, the bioactive peptides in oats still need to be further explored.

Therefore, in this study, we analyzed the physicochemical properties of oat peptides (OPs), determined the peptide composition, and predicted the functions of the OPs. The preventive effect of dietary supplementation with OPs on colitis was evaluated with a DSS-induced colitis model, and the anti-inflammatory and antioxidant activities of OPs were characterized. The regulatory effects of OPs on the intestinal flora were analyzed by 16S rRNA sequencing of intestinal microorganisms. Molecular docking was used to predict and preliminarily validate the potential targets by which OPs exert their protective effects in cells. The results provide a theoretical basis for the use of OPs as dietary supplements for the prevention of IBD.

## 2. Materials and Methods

### 2.1. Materials

Dextran sulfate sodium salt (DSS) (160110, MP Biomedicals, San Diego, CA, USA), total RNA extraction kit (Sevenbio, Beijing, China), reverse transcription kit (Monad, Jiangsu, China), SYBR qPCR fluorescent dyes (Monad, Suzhou, China), OPs purchased from Shanxi Ronglin Biotechnology Co. (Xian, China), Cell Counting Kit-8 (Life-iLab, Shanghai, China), ELISA kits for IL-1β, IL-6, and TNF-α (SINOBESTBIO, Shanghai, China), mouse superoxide dismutase (SOD) ELISA Kit (YX-E20348), mouse malondialdehyde (MDA) ELISA Kit (YX-E20347), total antioxidant capacity (T-AOC) Assay Kit (SINOBESTBIO, Shanghai, China), four peptides Trp-Gly-Val-Gly-Val-Arg-Ala-Glu-Arg-Asp-Ala (WGVGVRAERDA), Gln-Pro-Pro-Phe-Val-Gln-Gln-Glu-Gln-Pro(QPPFVQQEQP), Gln-Pro-Gln-Met-Gln-Gln-Gln-Phe-Phe-Gln-Pro-Gln (QPQMQQQFFQPQ), and Gln-Ala-Gly-Leu-Tyr-Phe-Leu (QAGLYFL) synthesized by GenScript Corporation (Nanjing, Nanjing, China). Cell culture-related supplies were purchased from Gibco (Grand Island, NY, USA) if not otherwise specified.

The following antibodies were used in this study: anti-Keap1 antibody (10503-2-AP, Proteintech, Chicago, IL, USA); anti-Nrf2 antibody (16396-1-AP, Proteintech, Chicago, IL, USA); anti-Muc2 antibody (DF8390, Affinity Biosciences, Cincinnati, OH, USA); anti-claudin 1 antibody (AF0127, Affinity Biosciences, Cincinnati, OH, USA); anti-ZO-1 antibody (AF5145, Affinity Biosciences, Cincinnati, OH, USA); anti-occludin antibody (DF7504, Affinity Biosciences, Cincinnati, OH, USA); anti-Alkaline phosphatase antibody (DF6225, Affinity Biosciences, Cincinnati, OH, USA); anti-β-actin antibody (4970, Cell Signaling Technology, Danvers, MA, USA); anti-GaPdh antibody (2118, Cell Signaling Technology, Danvers, MA, USA); anti-HO-1 antibody (A1346, ABclonal, Wuhan, China); anti-rabbit IgG antibody (7074, Cell Signaling Technology, Danvers, MA, USA).

### 2.2. Animals and Experimental Design

Twenty-four specific pathogen-free (SPF)-grade 6- to 7-week-old male BALB/c mice were purchased from Liaoning Changsheng Biotechnology Co. (SCXK (Liao) 2020-0001). After one week of acclimatization, they were randomly divided into 3 groups of 8 mice each: the negative control (NC) group, the DSS model group, and the DSS+OP treatment group. The experimental protocol was approved by the Animal Ethics and Welfare Committee of Jilin University (SY202305003), the experiments were carried out in the barrier facility of Jilin University Laboratory Animal Center (SYXK (Ji) 2021-0006), and the rearing conditions were strictly in accordance with GB14925 (Laboratory animal—Requirements of environment and housing facilities).

**Figure 1:**
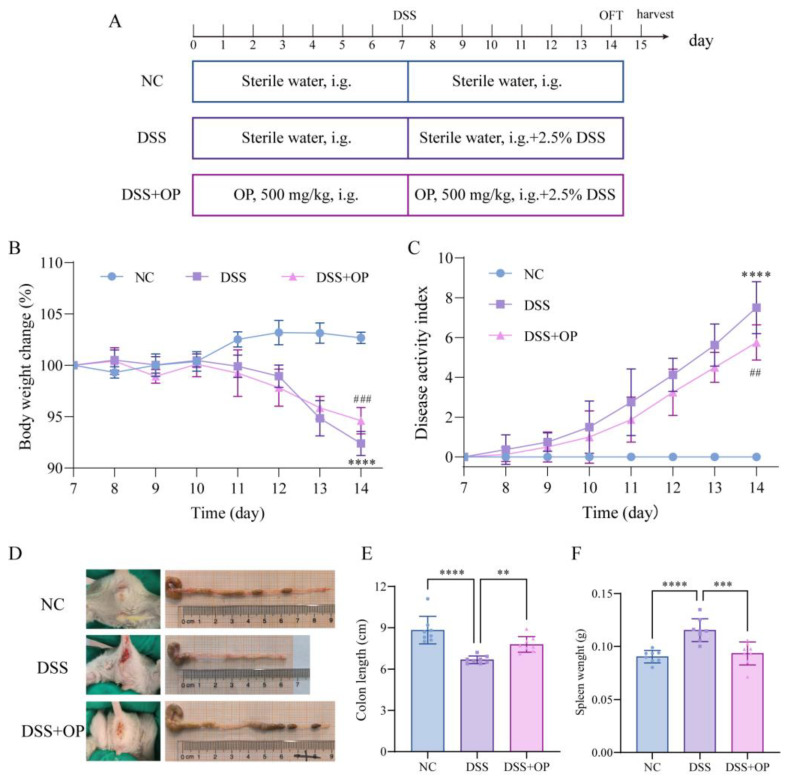
OPs alleviate DSS-induced colitis. (**A**) experimental protocol; (**B**) change in body weight; (**C**) DAI score; (**D**) representative photographs of perianal bleeding and colon tissues; (**E**) length of colon; (**F**) weight of spleen. Data are presented as mean ± SD (*n* = 8 per group). ** *p* < 0.01, *** *p* < 0.001, **** *p* < 0.0001; ## *p* < 0.01, ### *p* < 0.001 (* indicates comparison with the NC group in graphs (**B**,**C**), # indicates comparison with the DSS group in graphs (**B**,**C**).

The experimental procedure is shown in Figure 1A. The mice in the DSS+OP group were gavaged with 500 mg/kg OPs daily (peptide concentrations determined by pre-experimentation after reference to relevant literature), while mice in the NC and DSS groups were gavaged with equal amounts of saline. Acute colitis was induced by feeding distilled water containing 2.5% DSS to the DSS and DSS+OP groups starting on day 7 of the experiment. The mice in the NC group received only normal distilled water. The status of the mice was observed daily, and the disease activity index (DAI) [22] was scored according to the percentage of weight loss, fecal characteristics, and the degree of blood in the stool, as follows: DAI = (weight loss score + fecal trait score + fecal blood score) (Table S1).

Open field test (OFT) experiments were performed on day 14, and samples were collected for follow-up analysis on day 15.

### 2.3. Cell Culture

Caco-2 cells (CX0094, Boster Biological Technology Co., Ltd., Wuhan, China) were cultured in minimum essential medium (MEM) supplemented with 20% fetal bovine serum, and 1% double antibiotic (penicillin 100 U/mL, streptomycin 100 ug/mL). Cells were mycoplasma free. When the cells reached 80% confluence, they were digested using 0.25% trypsin-EDTA and passaged in a 1:3 ratio, and cells of the 21st–29th generation were used for experiments. The cells were treated with 50 µg/mL OPs or the four peptides for 36 h when they had grown to 60–70% confluence. A Cell Counting Kit-8 (CCK-8) assay was performed to assess cell viability.

### 2.4. Open Field Test (OFT)

The OFT was conducted in a 40 cm × 40 cm × 40 cm open field box. The entire field was divided into 4 × 4 blocks of 16 squares; the 4 blocks in the center were considered the central area, while the 12 blocks around the periphery were considered the peripheral area. At the beginning of the experiment, the mice were placed in the central area, and their activity behavior was recorded within 5 min. Before each new assay, the box was wiped with distilled water and alcohol to avoid interference of residual odor with subsequent experiments. After the experiment was completed, SMART 3.0 software (Panlab, Barcelona, Spain) was used for analysis. The main parameters analyzed included the distance of movement, mean speed, time spent moving in the central area, and number of passages in the central and peripheral areas.

### 2.5. Determination of the Molecular Weight and Amino Acid Composition of OPs

The molecular weight distribution of OPs was determined by Size Exclusion Chromatography (SEC) following a method reported previously [23]. A BioCore SEC-300 analytical column (7.8 mm × 300 mm) was used. The absorbance was monitored at 214 nm. Tyrosine (181 Da), bovine serum albumin (66,000 Da), cytochrome C (12,365 Da), IgG (150,000 Da), and IgM (750,000 Da) were used as the standards to establish a reference calibration curve. All solutions were mass spectrum grade.

The samples were prepared in accordance with the established protocol, wherein they underwent filtration through a 0.22 µm filter membrane and were subsequently analyzed using an automated amino acid analyzer (Hitachi, Tokyo, Japan). The detection of hydroxyproline and proline occurred at a wavelength of 440 nm, while the remaining amino acids were detected at 570 nm. All chemicals utilized were of analytical grade.

### 2.6. Determination of the Peptide Composition of OPs by UPLC-MS/MS

Samples were solubilized using 50 mM NH_4_HCO_3_, reduced, alkylated, and desalted. The samples were analyzed by ultra-performance liquid chromatography–mass spectrum/mass spectrum (UPLC-MS/MS) to obtain raw mass spectra, which were compared with the PEAKS Studio (10.6) database to obtain peptide identification results at Biotech-Pack Scientific Co. Peptide activity was predicted with the online tool PeptideRanker (http://distilldeep.ucd.ie/PeptideRanker/) (accessed on 30 September 2023). All solutions were mass spectrum grade.

### 2.7. ELISA

IL-1β, IL-6, and TNF-α levels in colon tissue and serum and SOD and MDA levels and T-AOC in colon tissue were measured according to the operating instructions of an ELISA kit (SINOBESTBIO, Shanghai, China).

### 2.8. Histopathology

Colon samples were fixed in 4% paraformaldehyde for 24 h. Paraffin embedding was performed according to the conventional procedure, and 5 µm paraffin sections were used for subsequent staining. Hematoxylin–eosin (HE) staining was performed to observe histopathological damage: inflammatory cell infiltration, mucosal edema, crypt swelling disruption, and epithelial cell damage [24]. Alcian blue (AB) and periodic acid–Schiff (PAS) staining was performed to observe cupped cells and mucus secretion [25]. The IHC-Toolbox plugin in ImageJ was utilized to analyze the positive sections of AB staining, PAS staining, and IHC staining.

### 2.9. Immunohistochemistry and Immunofluorescence (IF)

Immunohistochemistry was used to detect the protein expression of Muc2 in paraffin sections of colonic tissues. IF was used to detect the protein expression of Keap1 and Nrf2 in Caco-2 cells after 36 h of OPs or the four peptides treatment. The primary antibodies used were anti-Muc2 (1:500), anti-Keap1 (1:1000), and anti-Nrf2 (1:1000) antibodies. The secondary antibody was Cy3-labeled goat anti-rabbit IgG (1:1000). The nuclei were stained using DAPI.

### 2.10. RT-qPCR and Western Blotting (WB)

Total RNA was extracted from colon tissues using Total RNA Extraction Kit (Sevenbio, Beijing, China), and we analyzed the concentration and quality of RNA using NanoDrop 2000 (Thermo Fisher Scientific, Waltham, MA, USA). The 800 ng RNA was reverse transcribed into cDNA for quantitative detection. Amplification was then performed using a real-time fluorescence quantitative PCR instrument (Thermo Fisher Scientific, Waltham, MA, USA) following a two-step thermal cycling program (pre-denaturation, 95 °C, 10 min; denaturation, 95 °C, 10 s; annealing and extension, 60 °C, 30 s): a total of 40 cycles of denaturation, annealing and extension were performed; Finally, the dissolution curve was collected using the default program of the machine. The expression levels of the target genes were analyzed according to the 2^−ΔΔCT^ method using *Actb* as an internal reference. The primers used are shown in Table S2.

Western blotting was used to detect the expression of tight junction proteins in mouse colon tissues and the expressions of Keap1 and Nrf2 in Caco-2 cells after 36 h of Ops or the four peptides (50 µg/mL) treatment. The primary antibodies included anti-ZO-1 (1:1000), anti-claudin 1 (1:1000), anti-occludin (1:1000), anti-Keap1 (1:1000), anti-Nrf2 (1:1000), anti-HO-1 (1:1000), anti-Gapdh (1:2000), and anti-β-actin (1:2000) antibodies. The secondary antibody was goat anti-rabbit IgG (1:4000). The general flow of the experiment is as follows: Each 1 mg of colon tissue was homogenized by adding 15 µL of radioimmunoprecipitation assay (RIPA) (containing 1% phenylmethanesulfonyl fluoride). The resulting mixture was centrifuged at 12,000× *g* for 15 min at 4 °C. The protein concentration of the samples was determined using the bicinchoninic acid assay (BCA) protein quantification kit. The samples were then mixed with 5× loading buffer and denatured at 95 °C for 10 min. Proteins were separated using a 10% SDS-PAGE gel with a protein upload volume of 15 µg. The separated proteins were transferred to polyvinylidene difluoride (PVDF) membranes using the wet transfer method. The membranes were blocked for 30 min at room temperature using protein-free rapid-blocking solution. The primary antibody was then incubated with the membranes for 2 h at room temperature. After incubation, the membranes were washed four times with TBST for 8 min each time. Subsequently, the secondary antibody was incubated with the membranes for 1 h at room temperature. The membranes were again washed four times with TBST for 8 min each time. Finally, the image was visualized using ECL, and the bands were quantitatively analyzed using ImageJ software (version 2.0.0). The PVDF membrane used in Western blotting was purchased from Merck Millipore (Billerica, MA, USA), and the other reagents were purchased from Epizyme (Shanghai, China).

### 2.11. Microbial Composition Analysis by 16S rRNA Sequencing

Total DNA was extracted from cecal contents using an OMEGA Soil DNA Kit (M5635-02) (OmegaBio-Tek, Norcross, GA, USA). After passing the quality test, the V3+V4 region (variable regions on 16S rRNA) was amplified using PCR primers (PCR primer sequences: 338F, 5′-ACTCCTACGGGAGGCAGCA-3′; 806R, 5′-GGACTACHVGGGTWTCTAAT-3′). The PCR amplification products were detected by 2% agarose gel electrophoresis and recovered for purification. Subsequently, the bacterial 16S rRNA composition was sequenced with an Illumina NovaSeq sequencing platform. Subsequent data analysis was completed with the PANOMIX Cloud platform (PANXMIX, Suzhou, China). Principal Component Analysis (PCA), Principal Coordinate Analysis (PCoA), and Nonmetric Multidimensional Scaling (NMDS) are three classical downscaling methods used for analyzing the Beta diversity of gut microbial composition. These methods measure the similarity in microbial composition between samples based on the distance between points in the analyzed results. PCA downscales based on species richness, PCoA sorts based on the distance matrix to find the best eigenvalues, and NMDS is a nonlinear model that compensates for the limitations of PCA and PCoA [26,27,28,29]. By using these three methods together, a more comprehensive understanding of sample Beta diversity can be obtained. Another useful tool, LDA Effect Size (LEfSe), is employed to identify species that show significant differences in abundance between groups [30].

### 2.12. Determination of Short-Chain Fatty Acids (SCFAs)

The concentrations of SCFAs in cecal contents were determined using GC-MS. Briefly, a 100 mg sample was homogenized with 500 µL of deionized water, and the supernatant was centrifuged at 12,000 rpm at 4 °C for subsequent determination. GC analysis was performed on a TRACE 1300 gas chromatograph (Thermo Fisher Scientific, Waltham, MA, USA). The GC was fitted with an Agilent HP-INNOWAX capillary column (30 m × 0.25 mm ID × 0.25 μm), and helium was used as the carrier gas at 1 mL/min. Injection was made in split mode at 10:1 with an injection volume of 1 μL and an injector temperature of 250 °C. Mass spectrometric detection of metabolites was performed on an ISQ 7000 (Thermo Fisher Scientific, Waltham, MA, USA) in electron impact ionization mode. Single ion monitoring (SIM) mode was used with an electron energy of 70 Ev [31,32].

### 2.13. Molecular Docking

The four peptides WGVGVRAERDA, QPPFVQQEQP, QPQMQQQFFQPQ, and QAGLYFL (later using the one-letter code for amino acids) were modeled using maestro software (version 11.9). The Keap1 protein was designated as the receptor, and the 4 peptides were designated as ligands. The 4 peptides were docked with the Keap1 protein (PDB ID: 2FLU) using MOE software (version 2015.10). The scores of the docking combinations of the 4 peptides with the Keap1 protein were calculated separately, and force analysis and visualization were performed using PyMOL (version 2.3) and Ligplus (version 2.2) software.

### 2.14. Statistical Analysis

The experimental data are presented as the mean ± standard deviation (SD). Unpaired *t*-tests were used to analyze differences between two groups, along with one-way ANOVA followed by Dunnett post-testing was used to compare multiple groups. The data were analyzed and plotted using GraphPad Prism9.5 (La Jolla, CA, USA). All experiments were repeated at least three times, and *p* < 0.05 indicated that the differences were statistically significant.

## 3. Results

### 3.1. OPs Alleviate the Symptoms of DSS-Induced Colitis in Mice

Before analyzing the effects of OPs, we characterized their physicochemical properties. The molecular weights of the OPs were determined through the utilization of Size Exclusion Chromatography (SEC). The obtained results indicated that the OPs were predominantly composed of peptides with molecular weights below 5 kDa. In particular, peptides with molecular weights of 1–5 kDa, 0.5–1 kDa and <500 Da accounted for 50.92%, 28.25%, and 15.87% of the total content, respectively (Table 1). Glutamate and glycine were the two most abundant amino acids in OPs (Table S3). Subsequently, using a model of DSS-induced acute colitis, we evaluated the alleviating effect of prophylactic OP supplementation on colitis. The results of animal experiments showed that after 7 days of DSS treatment, the mice in the DSS model group had significantly reduced body weights (Figure 1B), significantly elevated DAI scores (Figure 1C), severe perianal bleeding (Figure 1D), shortened colons (Figure 1E), and increased spleen weights (Figure 1F). In contrast, OP intervention significantly improved the aforementioned lesions.

### 3.2. OPs Alleviate Inflammation and Oxidative Stress in Mice with Colitis

**Figure 2:**
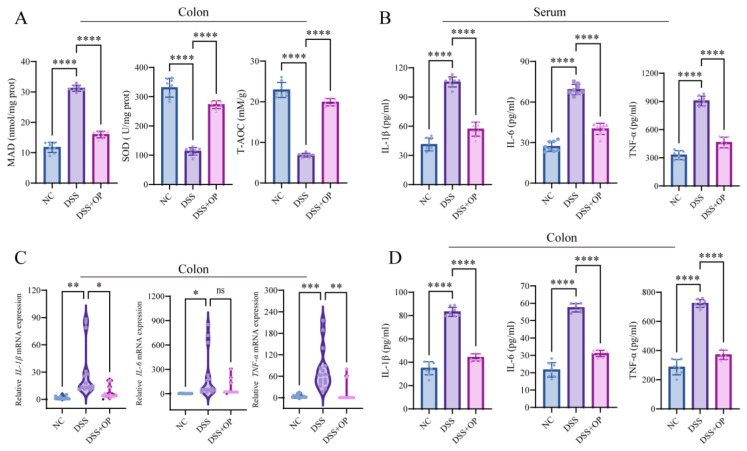
OPs inhibit inflammation and oxidative stress. (**A**) MDA level, SOD level, and T-AOC in the colon; (**B**) levels of the inflammatory factors IL-6, IL-1β, and TNF-α in serum; (**C**) mRNA expression levels of IL-6, IL-1β, and TNF-α in the colon; (**D**) levels of IL-6, IL-1β, and TNF-α in the colon. Data are presented as mean ± SD (A, B, D) (*n* = 8 per group). * *p* < 0.05, ** *p* < 0.01, *** *p* < 0.001, **** *p* < 0.0001, ns *p* > 0.05.

Colitis is often accompanied by an increased inflammatory response and oxidative damage [22,33]. Therefore, we assessed the effects of OPs on inflammation and oxidative stress in mice with colitis by measuring the levels of inflammatory markers (IL-6, IL-1β, TNF-α) and redox markers (MDA, SOD, T-AOC) in serum and colonic tissues. The results showed that the colon MDA content was significantly higher (*p* < 0.0001) and that the colon SOD content and T-AOC were significantly lower (*p* < 0.0001) in the DSS group than in the NC group, indicating that significant oxidative stress occurs in DSS-induced colitis. This oxidative stress was significantly alleviated by OPs treatment, as evidenced by significantly lower MDA levels (*p* < 0.0001) and significantly higher SOD levels and T-AOC values (*p* < 0.0001) in the DSS+OP group than in the DSS group (Figure 2A). Similarly, the levels of inflammatory factors in mouse colonic tissue and serum were measured by RT-qPCR and ELISA, which showed that OP intervention significantly inhibited the expressions of IL-6, IL-1β, and TNF-α (although it did not significantly alter the mRNA expression of IL-6 in the colon) (Figure 2B–D). The results described above suggest that OPs have inhibitory effects on inflammation and oxidative stress in mice with DSS-induced colitis.

### 3.3. OPs Repair the Intestinal Mechanical Barrier

**Figure 3:**
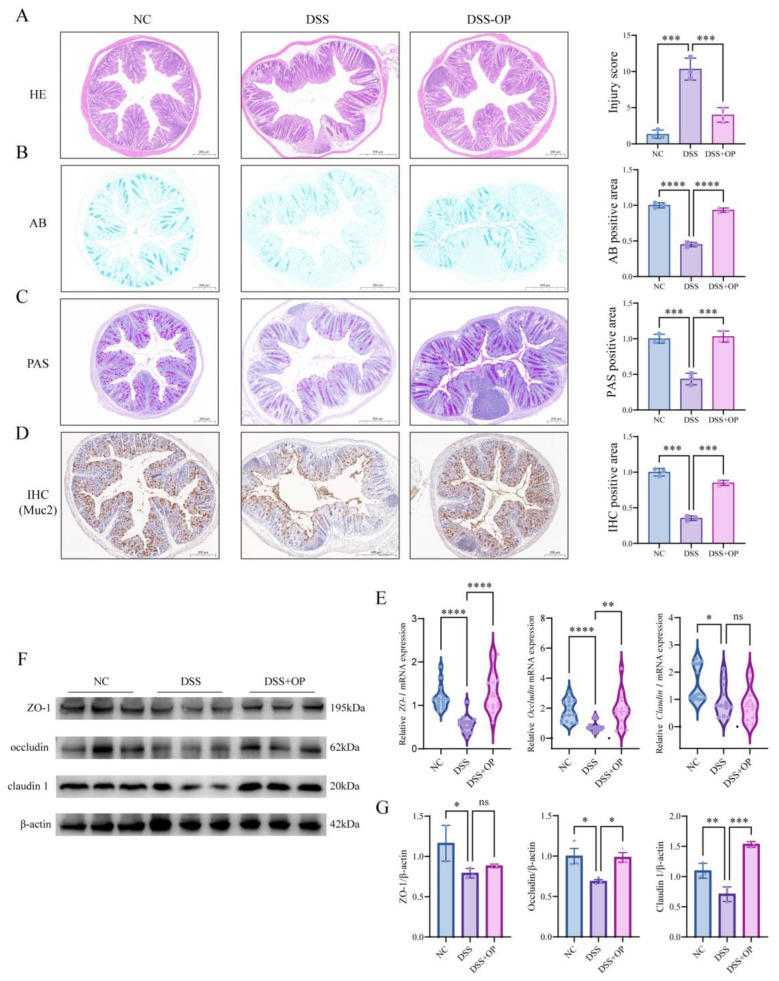
OPs repair the intestinal mechanical barrier. (**A**) HE staining results of colon tissue; (**B**) AB staining results; (**C**) PAS staining results; (**D**) IHC results for Muc2 protein; (**E**) mRNA expression of ZO-1, occludin and claudin 1; (**F**) Western blotting results for ZO-1, occludin and claudin 1; (**G**) normalized analysis results for the ZO-1, occludin and claudin 1 proteins. Data are presented as mean ± SD (**A**–**D**,**G**) (*n* = 3–5 per group). * *p* < 0.05, ** *p* < 0.01, *** *p* < 0.001, **** *p* < 0.0001, ns *p* > 0.05.

Tight junction proteins, cupped cells, and their secreted mucus together form the mechanical barrier of the intestine, which is the first line of defense against colitis [34]. In the next part of the experiment, we analyzed the effect of OPs on the intestinal mechanical barrier. The results showed that the colonic tissue was severely damaged after DSS treatment, as indicated by mucosal edema and inflammatory cell infiltration (Figure 3A), reduced mucus secretion (Figure 3B), and reduced cup cell numbers (Figure 3C,D). However, OP intervention significantly ameliorated these pathological changes; in addition, we detected changes in the expression of tight junction proteins (Muc2, ZO-1, occludin, and claudin 1) in the colon via IHC, RT-qPCR, and Western blotting. In particular, the expression levels of tight junction proteins were significantly reduced after DSS treatment. In contrast, the mRNA expression levels of ZO-1 and occludin were significantly higher in the DSS+OP group than in the DSS group (*p* < 0.01) (Figure 3E), as were the protein expression levels of occludin and claudin 1 (*p* < 0.05) (Figure 3G). The above results suggest that OPs can repair the intestinal mechanical barrier by promoting the expression of tight junction proteins and facilitating mucus secretion.

### 3.4. OPs Alleviate DSS-Induced Behavioral Disorders

**Figure 4:**
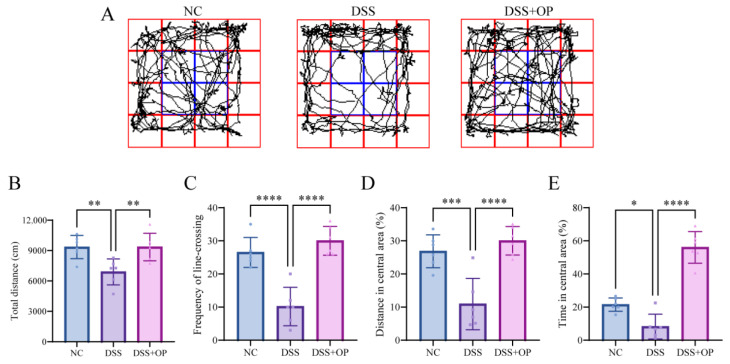
OPs alleviate DSS-induced behavioral disorders. (**A**) Representative images from the OFT; (**B**) total distance of movement; (**C**) number of cross-region traversals; (**D**) distance of movement in the central area; (**E**) dwell time in the central area. Data are presented as mean ± SD (*n* = 6 per group).* *p* < 0.05, ** *p* < 0.01, *** *p* < 0.001, **** *p* < 0.0001.

Several studies have shown depression and anxiety-like manifestations to be comorbidities of colitis [35,36,37]. Additionally, supplementation with oat extract has been reported to benefit cognitive function and modulate physiological responses to stress [38]. Therefore, via an OFT, we analyzed the protective effect of OPs against DSS-induced behavioral disorders in mice. Figure 4A shows the representative images of the motor trajectories of mice in the three groups. The analysis showed that compared to the mice in the NC group, the mice in the DSS group traveled significantly shorter total distances (*p* < 0.01) (Figure 4B), traveled between the central and peripheral regions significantly fewer times (*p* < 0.0001) (Figure 4C), traveled significantly shorter distances in the central region (*p* < 0.001) (Figure 4D), and spent significantly less time in the central region (*p* < 0.05) (Figure 4E). In contrast, all indicators were significantly improved in the DSS+OP group. The above results suggest that OPs have significant alleviating effects on DSS-induced behavioral disorders.

### 3.5. OPs Regulate the Production of SCFAs

**Figure 5:**
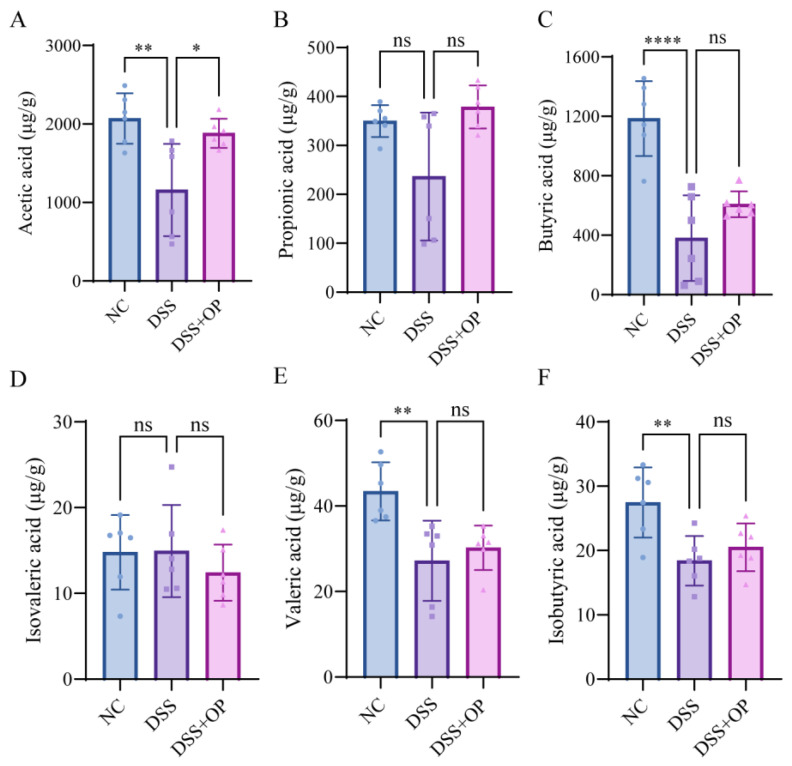
OPs regulate the production of SCFAs. (**A**–**F**) Acetic acid, propionic acid, butyric acid, butyric acid, isovaleric acid, valeric acid, and isobutyric acid. Data are presented as mean ± SD (*n* = 6 per group). * *p* < 0.05, ** *p* < 0.01, **** *p* < 0.0001, ns *p* > 0.05.

SCFAs are produced by bacterial fermentation in the intestine and exert a variety of effects on host metabolism and the immune system [39]. SCFAs are important signaling molecules for the function of intestinal microorganisms [39]. The results of SCFA content measurement in mouse ceca are shown in Figure 5. The quantities of acetic acid, butyric acid, isobutyric acid, valeric acid, and isovaleric acid were decreased significantly (*p* < 0.01) in the DSS group, while the changes in the quantities of propionic acid and isovaleric acid were not significant. The content of acetic acid in the DSS+OP group was significantly higher than that in the DSS group (*p* < 0.05), and the content of other short-chain fatty acids was not significantly different from that in the DSS group.

### 3.6. OPs Alleviate DSS-Induced Intestinal Bacterial Disorders

**Figure 6:**
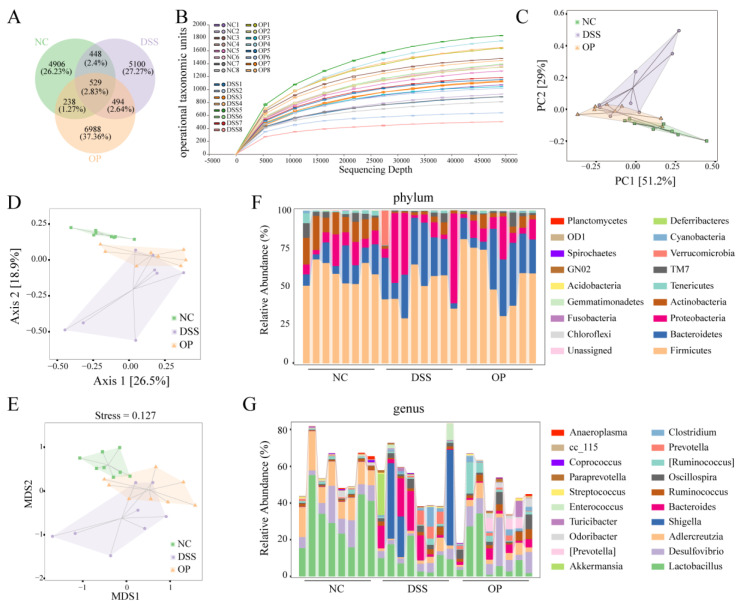
OPs alleviate DSS-induced intestinal bacterial disorders. (**A**) Venn diagram showing the distribution of ASVs; (**B**) rarefaction curve; (**C**) PCA; (**D**) PCoA; (**E**) NMDS analysis; (**F**) microbial composition of each group at the phylum level (TM7 indicates Saccharibacteria; GN02 indicates Gracilibacteria; OD1 indicates Parcubacteria) (each column represents one mouse sample); (**G**) microbial composition of each group at the genus level ([Ruminococcus] refers to unidentified strains of the enteric flora that may belong to the genus Ruminococcus) (each column represents one mouse sample). (*n* = 8 per group).

The regulatory effects of OPs on intestinal microbes were analyzed via 16S rRNA sequencing. The Venn diagram demonstrates the distribution of amplicon sequence variants (ASVs) in the three groups (NC, DSS, and DSS+OP) (Figure 6A). The sparse plot proved that the amount of sequencing data was sufficient and reflected the trend of reduced α diversity in the DSS group (Figure 6B). However, the Chao1 and Shannon index analysis results showed no significant effect of OP intervention on α diversity (Figure S1). Cluster analysis using PCA, PCoA, and NMDS showed that the gut microbial composition of the DSS group significantly deviated from that of the NC group but that OP intervention regressed the microbial composition toward that of the NC group (Figure 6C–E). The compositions of microbes at the phylum and genus levels are shown in Figure 6F,G. Compared to the NC group, the DSS group had greater abundance of Proteobacteria and Actinobacteria but lower abundance of Firmicutes at the phylum level, while it had greater abundance of Shigella and Bacteroides but lower abundance of Lactobacillus and Desulfovibrio at the genus level. In contrast, OP intervention had an alleviating effect on DSS-induced intestinal bacterial disorders such that the composition of the DSS+OP group was more similar to that of the NC group than that of the DSS group.

**Figure 7:**
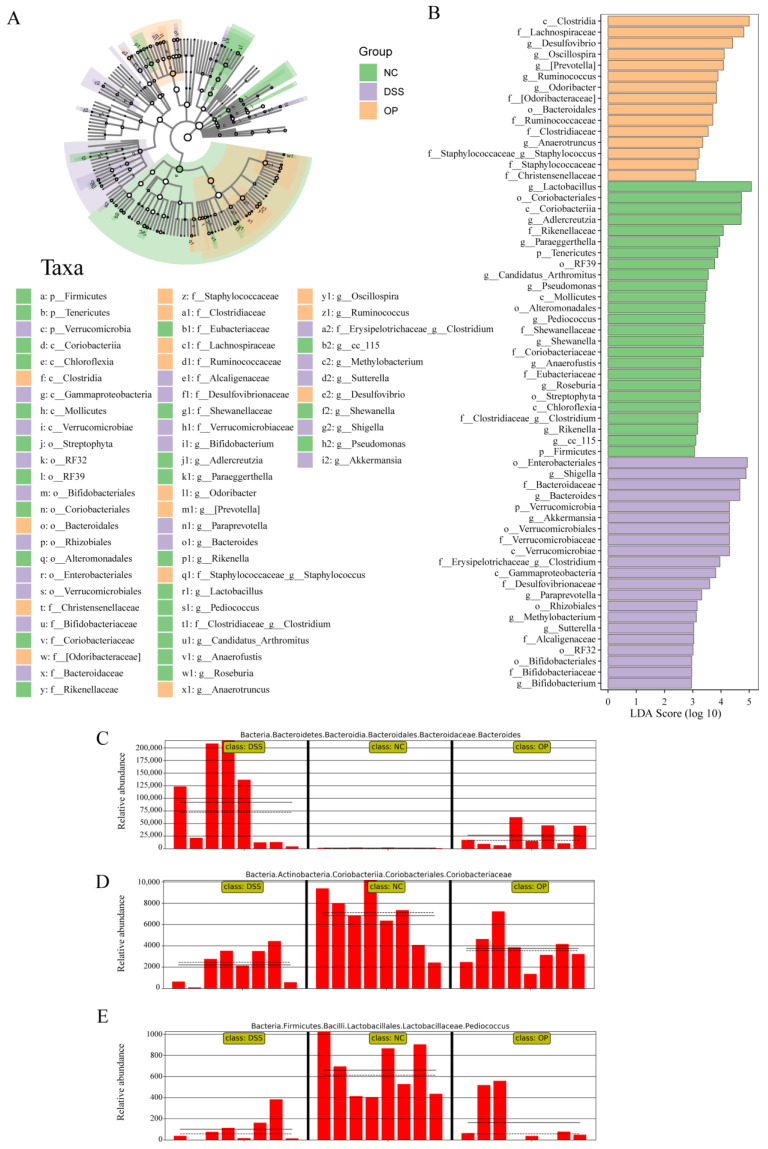
LEfSe analysis revealed differential microbes between groups. (**A**) Cladogram from LEfSe analysis; (**B**) LDA scores in differentially abundant taxa (LDA > 2.0) (*p*, c, o, f, g indicate taxonomic boundaries of microorganisms corresponding to phylum, class, order, family, and genus, respectively); (**C**–**E**) relative abundance of Bacteroides, Coriobacteriaceae, and Pediococcus in each sample (the solid and dashed lines identify the mean and median relative abundance of this taxonomic unit in each grouping). (The headings on the horizontal axes C, and E indicate attribution at the kingdom, phylum, class, order, family, and genus taxonomic levels, respectively; the headings on the horizontal axes D indicate attribution at the kingdom, phylum, class, order, and family taxonomic levels, respectively). (*n* = 8 per group).

The microbes that differed between groups were further analyzed by LEfSe. The cladogram demonstrates the taxonomic hierarchy of marker species in each group of samples (Figure 7A), and the histogram of the distribution of LDA values demonstrates the species that were significantly enriched within the three groups (Figure 7B). The results showed that the abundance of Bacteroides increased (Figure 7C) and that the abundance of Coriobacteriaceae and Pediococcus decreased (Figure 7D,E) after DSS induction, while the abundance of the corresponding bacteria was restored toward that in the NC group after OPs intervention. The above results indicate that OPs can alleviate DSS-induced intestinal flora disorder.

**Figure 8:**
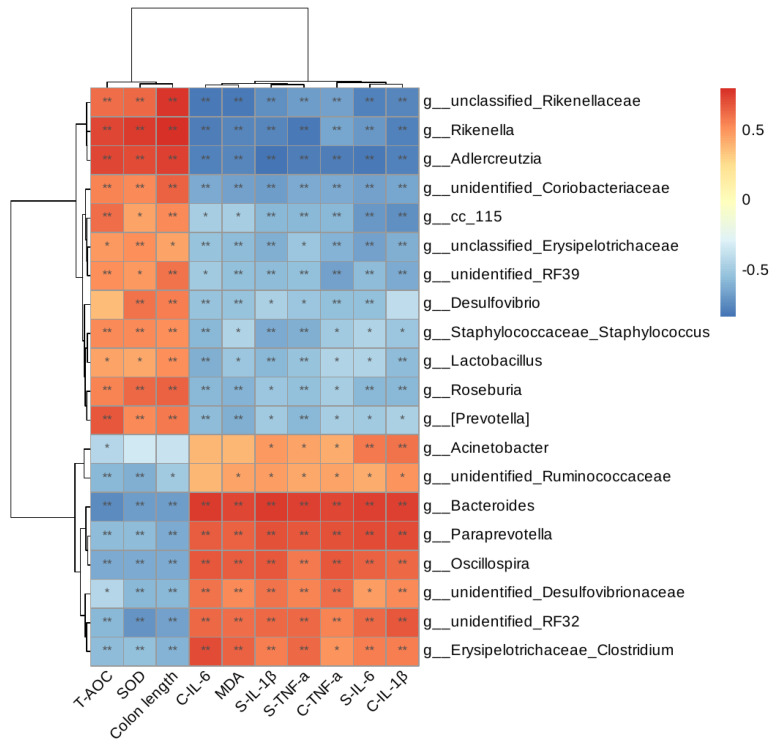
Results of Spearman correlation analysis between biochemical indicators and intestinal microbes. (The S-prefix indicates the amount in serum and the C-prefix indicates the amount in colon tissue) * *p* < 0.05, ** *p* < 0.01.

Spearman correlation analysis was employed to investigate the association between biochemical indicators and gut microbiota (Figure 8). The findings revealed that Rikenella, Adlercreutzia, Desulfovibrio, and Staphylococcaceae exhibited negative correlations with pro-inflammatory factors while displaying positive correlations with T-AOC, SOD, and colon length. Conversely, Bacteroides, Paraprevotella, and Oscillospira demonstrated positive correlations with pro-inflammatory factor levels, while exhibiting negative correlations with T-AOC, SOD, and colon length.

### 3.7. Screening of Bioactive Peptides in OPs and Prediction of Target Proteins via Molecular Docking

To further analyze the potential bioactive peptides in OPs, the peptide composition of OPs was identified based on UPLC-MS/MS, and a total of 266 peptide sequences were obtained (Table S4). The activity of the peptides was predicted with the PeptideRanker scores database. Information about the top 15 peptides in the content ranking is shown in Table 2, including the peptide sequence, molecular weight, and predicted activity. Considering the bioactivity and abundance of the peptides, WGVGVRAERDA, QPPFVQQEQP, QPQMQQQFFQPQ, and QAGLYFL were selected for subsequent analysis.

The Keap1-Nrf2 signaling axis plays a key role in the maintenance of redox homeostasis in cells and regulates the inflammatory response [40]. Within this axis, Keap1 is an endogenous inhibitor of Nrf2. In the resting state, Keap1 binds to Nrf2 to confine it to the cytoplasm and promote its degradation via the proteasome pathway; however, in the presence of oxidative stress, Nrf2 separates from Keap1 and acts in the nucleus [41,42]. Based on the regulatory effects of OPs on inflammation and oxidative stress, we speculated that OPs could bind Keap1 and deregulate the effect of Keap1 on Nrf2. The molecular docking results suggest that bioactive peptides in OPs may have binding potential to Keap1. Specific molecular docking results are shown in Figure S2 and Table S5.

### 3.8. OPs Regulate the Keap1-Nrf2 Signaling Axis in Caco-2 Cells

**Figure 9:**
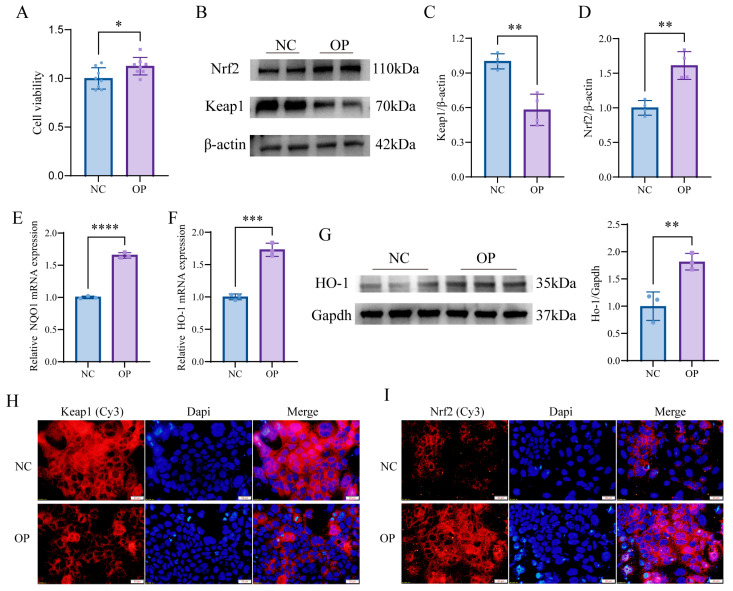
OPs regulate the Keap1-Nrf2 signaling axis. (**A**) CCK-8 assay of the effect of OPs on cell proliferation; (**B**–**D**) Western blotting assay results for Keap1 and Nrf2; (**E**,**F**) mRNA expression of NQO1 and HO-1; (**G**) Western blotting assay results for HO-1; (**H**,**I**) immunofluorescence assay results for Keap1 and Nrf2 in Caco-2 cells after OPs treatment. Data are presented as mean ± SD (*n* = 3–8 per group). * *p* < 0.05, ** *p* < 0.01, *** *p* < 0.001, **** *p* < 0.0001.

To further test the above hypothesis, we treated Caco-2 cells with 50 µg/mL OPs for 36 h and analyzed the changes in Keap1 and Nrf2 protein expression levels and expression locations by immunofluorescence and Western blotting. A CCK-8 assay showed no cytotoxicity of OPs (Figure 9A), and Western blotting, RT-qPCR, and immunofluorescence assays showed that after OP treatment, Keap1 protein expression was decreased (Figure 9B,D,H), Nrf2 protein expression was increased, Nrf2 nuclear localization signals were enhanced (Figure 9B,C,I), and Nrf2 downstream targets NQO1 and HO-1 were activated (Figure 9E–G).

Cells were subjected to treatment with four synthesized peptides, namely WGVGVRAERDA (OP1), QPPFVQQEQP (OP2), QPQMQQQQFFQPQ (OP3), and QAGLYFL (OP4), in order to assess the expression of the Keap1-Nrf2 signaling axis. The findings indicated that OP1, OP2, and OP4 exhibited significant inhibitory effects on Keap1 expression, and OP3 had no significant effect on the protein expression of Keap1 (Figures S3 and S4). In addition, the results of immunofluorescence staining showed that OP1 facilitated the translocation of Nrf2 into the nucleus, whereas OP2, OP3, and OP4 had no significant effect on the localization of Nrf2 expression (Figure S5). These results suggest that WGVGVRAERDA may serve as the principal constituent responsible for the functionality of the OPs.

## 4. Discussion

UC is an inflammatory disease that occurs mainly in the colon and rectum [43]. Since the middle of the last century, the incidence of UC has increased yearly, imposing a very large burden on public health [1,44]. The animal model of DSS-induced colitis has clinical manifestations (weight loss, blood changes, and fecal irregularities) and pathological features (intestinal barrier damage, increased oxidative stress and proinflammatory response, etc.) similar to those of UC patients [45]. Studies based on this model have shown that foxtail millet peptides produced by protein hydrolysis can alleviate DSS-induced colitis in mice by inhibiting NLRP3 inflammatory vesicle formation and Th17 cell differentiation [46]. Acidic Mammalian Chitinase Inhibitor OAT-177 alleviates DSS-induced injury by exerting anti-inflammatory effects [47]. In addition, rice protein peptides can alleviate DSS-induced colitis by modulating intestinal flora and modulating Keap1-Nrf2 [23,48]. In addition, prophylactic supplementation with wheat germ-derived hybrid peptides (520 mg/kg), as well as APEPEPAF (50 mg/kg), was able to alleviate DSS-induced colitis by inhibiting the NF-κB pathway and ameliorating gut microbiota disturbances [49]. These studies suggest that food-borne peptides have a greater potential for use as dietary supplements in the prevention of UC.

In this study, we first assessed the physicochemical properties of OPs, and the results showed that most OPs have molecular weights below 5 kDa; such small peptides are more easily absorbed by the organism than larger peptides. In the DSS-induced acute colitis mouse model, OP intervention effectively alleviated colitis-related symptoms and suppressed inflammation and oxidative stress. The molecular weight of peptides is an important factor affecting their activity. Zishan Hong et al. focused on small molecular weight peptides below 1 kDa and found that dietary supplementation with three peptides produced by the enzymatic digestion of walnut proteins (SHTLP, HYNLN, and LGTYP) proved to ameliorate DSS-induced UC by inhibiting the activation of the TLR4-MAPK pathway and alleviating gut microbial disturbances, with the three peptides supplemented at a dose of 800 mg/kg [50]. To clarify the underlying mechanism of its effects, we identified the peptide composition of OPs based on UPLC-MS/MS. Among them, WGVGVRAERDA is a peptide with the highest percentage of content in OPs. In the Caco-2 cell model, OP and WGVGVRAERDA treatment decreased Keap1 protein expression, increased Nrf2 protein expression, and significantly induced the nuclear translocation of the Nrf2 protein. In contrast, QPPFVQQEQP and QAGLYFL had some inhibitory effects on Keap1 protein expression, but none of the three peptides, QPPFVQQEQP, QAGLYFL, and QPQMQQQQQQFFQPQ, was able to activate Nrf2. These findings suggest that OPs may exert anti-inflammatory and antioxidant effects by regulating the Keap1-Nrf2 signaling axis, and WGVGVRAERDA may be the main bioactive peptide in OPs.

The intestinal barrier mainly consists of mechanical barriers (tight junction proteins, mucus layer, and cupped cells), microbial barriers, and immune barriers. In the present study, we analyzed the protective effects of OPs on mechanical and microbial barriers. OPs were able to repair the intestinal mechanical barrier by promoting tight junction protein expression and mucus secretion. In addition, OPs were able to increase the abundance of Coriobacteriaceae and Pediococcus while decreasing the abundance of Bacteroides. The function of Coriobacteriaceae is associated with SCFA synthesis [51,52], and Coriobacteriaceae abundance is significantly reduced in IBD [53]. *Pediococcus* is a member of the Lactobacillus family with therapeutic effects on inflammation, fatty liver, and obesity [54]. Some studies have confirmed the alleviating effect of the transplantation of *Pediococcus pentosaceus* on DSS-induced colitis [55,56]. Bacteroides are an important group of pathogenic bacteria in the intestine, and in IBD, the abundance of enterotoxigenic *Bacteroides fragilis* (ETBF) is elevated [57]. It has been demonstrated that ETBF promotes intestinal inflammation and malignancy by disrupting intercellular communication in the host [58]. In this study, the abundance of *Coriobacteriaceae* and *Pediococcus* increased, and *Bacteroides* decreased after OP intervention. These results suggest that OPs can alleviate intestinal bacterial imbalance by decreasing the abundance of harmful bacteria and increasing the abundance of beneficial bacteria.

Oat beta-glucan, a non-starch polysaccharide found in the endosperm and dextrin layer of oats, cannot be digested or absorbed by the stomach or small intestine. Functioning as a prebiotic, oat beta-glucan is primarily metabolized by bacteria in the large intestine, thereby positively impacting the composition of the intestinal microbiota. A study conducted by Junying Bai et al. further confirmed the therapeutic potential of oat beta-glucan in alleviating colitis through its modulation of the intestinal flora [16]. In contrast, it is noteworthy that OP can be readily absorbed by the body without the need for digestion, rendering it a more suitable dietary supplement than oat beta-glucan for individuals with poor digestion and weakened constitution. Furthermore, peptides exhibit a diverse range of biological activities, and our current investigation has revealed that OP possesses the ability to elicit anti-inflammatory and antioxidant effects by selectively modulating the Keap1-Nrf2 signaling axis. Consequently, OP demonstrates a wider range of potential applications. Nevertheless, it is important to acknowledge that both OP and β-glucan are functionally active constituents of oats, the results of these studies will provide essential information for the application of oats.

When colitis occurs, damage to the intestinal barrier leads to increased intestinal permeability and disturbances in the metabolism of the organism [59,60]. Secondarily, comorbidities such as liver damage, metabolic diseases, and nerve damage arise [61,62,63]. OPs showed excellent performance in alleviating DSS-induced behavioral disorders, as they significantly increased the movement distance and exploratory behavior in the DSS+OP group of mice. This suggests that we can focus on the anti-fatigue and neuroprotective functions of OPs in the future.

Notably, there were some limitations in this study. For example, the regulatory effects of OPs on the immune barrier have not been explored. Although cellular experiments demonstrated the regulatory effects of WGVGVRAERDA on the Keap1 and Nrf2 proteins. However, the therapeutic efficacy of WGVGVRAERDA for IBD still needs further evaluation in animal experiments. In addition, the molecular weight of peptides is an important factor affecting their activity, and a comparison of the effects of different enzymatic methods on the composition and activity of peptides from oat sources could be considered. This could further explore the nutritional value of oats and develop efficient and safe IBD prevention supplements.

## 5. Conclusions

Overall, this study shows that OPs alleviate DSS-induced colitis by maintaining the intestinal barrier and modulating the Keap1-Nrf2 axis. The findings provide a theoretical basis for the use of OPs as dietary supplements for the prevention of IBD.

## Figures and Tables

**Table 1:** Molecular weight of OPs.

No.	Molecular Weight Distribution	Percentage (%)
1	>10 kDa	1.02
2	5–10 kDa	3.94
3	1–5 kDa	50.92
4	0.5–1 kDa	28.25
5	<500 Da	15.87

**Table 2:** Potential bioactive peptides in OPs.

No.	Peptide	Length	Mass (Da)	Error (ppm)	Experimental *m/z*	Retention Time (min)	Peptide Ranker Score ^#^	Percentage of Ops (%)
1	WGVGVRAERDA	11	1214.62	−3.2	608.32	21.46	0.15	33.55%
2	QPPFVQQEQP	10	1196.58	6	599.31	96.73	0.22	18.19%
3	QPQMQQQFFQPQ	12	1533.70	7.5	767.87	86.7	0.36	5.33%
4	STPAPAPAPA	10	878.45	−0.2	440.23	24.19	0.54	4.05%
5	GLVQPQTQMAGQVFIQPQQLAQYQAMKVVAMQT	33	3733.86	0.3	934.48	27.07	0.02	3.70%
6	QPQLQQVFNQPQ	12	1495.74	−7.8	748.88	72.63	0.21	3.65%
7	QMGLVQPQTQMASQVFIQPQQLPQYQA	27	3086.53	3.3	618.32	20.21	0.11	2.76%
8	QPPFVQQEQPFVQQQ	15	1826.90	−6.4	914.45	43.21	0.19	2.74%
9	QAGLYFL	7	852.44	6.6	427.23	12.02	0.84	2.30%
10	EFPLGYKTFGEAIPPQ	16	1792.90	−8.4	897.46	104.62	0.48	2.17%
11	QVSQPQLQLQQQVFQPQ	17	2065.06	9.6	1033.55	57.25	0.15	1.78%
12	PQLQQVFNQPQ	11	1325.67	4	663.85	78.7	0.19	1.53%
13	EQQQSILQQQQMLLQQQQQMLL	22	2785.37	−2.3	697.35	35.86	0.14	1.28%
14	AGERPEEAAVQPQ	13	1422.67	7.9	712.35	35.11	0.10	1.00%
15	HYINNSQALRSGI	13	1471.75	1.7	368.95	17.66	0.17	0.86%

Peptide Ranker score ^#^: Scoring of peptide activity using the PeptideRanker website (http://distilldeep.ucd.ie/PeptideRanker/) (accessed on 30 September 2023).

## Data Availability

The raw sequence data reported in this paper have been deposited in the Genome Sequence Archive in the National Genomics Data Center, China National Center for Bioinformation/Beijing Institute of Genomics, Chinese Academy of Sciences (GSA: CRA011356) are publicly accessible at https://ngdc.cncb.ac.cn/gsa/browse/CRA011356 (accessed on 8 July 2023).

## Supplementary Materials

The following supporting information can be downloaded at: https://www.mdpi.com/article/10.3390/nu15245055/s1, Figure S1. Results of alpha diversity analysis; Figure S2. Molecular docking prediction of the interactions of four bioactive peptides with the receptor protein Keap1; Figure S3. Western blotting analysis of the effect of four peptides on Keap1; Figure S4. Immunofluorescence analysis of the effect of four peptides on Keap1; Figure S5. Immunofluorescence analysis of the effect of four peptides on Nrf2; Table S1. Amino acid composition of OPs; Table S2. The assessment criteria for DAI scoring; Table S3. Primers of RT-qPCR; Table S4. Composition of peptides in OPs; Table S5. MOE Score, Number of formed hydrogen bonds, and Amino acid residues of peptides in OPs for docking with the Keap1 protein.

## Abbreviations

AB	alcian blue
ASV	amplicon sequence variants
CD	Crohn’s disease
CCK-8	Cell Counting Kit-8
DAI	disease activity index
DSS	dextran sulfate sodium salt
ELISA	enzyme-linked immunosorbent assay
ETBF	Enterotoxigenic Bacteroidesfragilis
HE	hematoxylin–eosin
IBD	inflammatory bowel disease
IF	immunofluorescence
IHC	immunohistochemistry
LEfSe	LDA Effect Size
MDA	malondialdehyde
MEM	minimum essential medium
MS	mass spectrum
Muc2	mucin 2
NMDS	nonmetric multidimensional scaling
OFT	open field test
OPs	oat peptides
OTU	operational taxonomic units
PAS	periodic acid-Schiff
PCA	principal component analysis
PCoA	principal coordinate analysis
PVDF	polyvinylidene difluoride
RT-qPCR	real-time quantitative PCR
SCFA	short-chain fatty acid
SEC	size exclusion chromatography
SOD	superoxide dismutase
SPF	specific pathogen free
T-AOC	total antioxidant capacity
TNBS	trinitrobenzene sulfonic acid
Tregs	regulatory T cells
UC	ulcerative colitis
UPLC	ultra-performance liquid chromatography
WB	Western blotting

## References

[1] Kaplan G.G. (2015). The global burden of IBD: From 2015 to 2025. Nat. Rev. Gastroenterol. Hepatol..

[2] Ramos G.P., Papadakis K.A. (2019). Mechanisms of Disease: Inflammatory Bowel Diseases. Mayo Clin. Proc..

[3] Panaccione R., Danese S., Zhou W., Klaff J., Ilo D., Yao X., Levy G., Higgins P.D.R., Loftus E.V., Chen S. (2023). Efficacy and safety of upadacitinib for 16-week extended induction and 52-week maintenance therapy in patients with moderately to severely active ulcerative colitis. Aliment. Pharmacol. Ther..

[4] Sandborn W.J., D’Haens G.R., Sands B.E., Panaccione R., Ng S.C., Lawendy N., Kulisek N., Modesto I., Guo X., Mundayat R. (2023). Tofacitinib for the Treatment of Ulcerative Colitis: An Integrated Summary of up to 7.8 Years of Safety Data from the Global Clinical Programme. J. Crohns Colitis.

[5] Peyrin-Biroulet L., Vermeire S., D’Haens G., Panes J., Dignass A., Magro F., Nazar M., Le Bars M., Lahaye M., Ni L. (2023). Clinical trial: Clinical and endoscopic outcomes with ustekinumab in patients with Crohn’s disease: Results from the long-term extension period of STARDUST. Aliment. Pharmacol. Ther..

[6] deBruyn J.C., Huynh H.Q., Griffiths A.M., Jacobson K., Mack D., Deslandres C., El-Matary W., Otley A.R., Church P.C., Lawrence S. (2023). Adalimumab Versus Infliximab in Luminal Pediatric Crohn’s Disease: Comparable Outcomes in a Prospective Multicenter Cohort Study. Am. J. Gastroenterol..

[7] Martinez-Villaluenga C., Penas E. (2017). Health benefits of oat: Current evidence and molecular mechanisms. Curr. Opin. Food Sci..

[8] Chu Y.F., Wise M.L., Gulvady A.A., Chang T., Kendra D.F., Jan-Willem van Klinken B., Shi Y., O’Shea M. (2013). In vitro antioxidant capacity and anti-inflammatory activity of seven common oats. Food Chem..

[9] Chen C.Y., Milbury P.E., Collins F.W., Blumberg J.B. (2007). Avenanthramides are bioavailable and have antioxidant activity in humans after acute consumption of an enriched mixture from oats. J. Nutr..

[10] Chen C., Wang L., Wang R., Luo X., Li Y., Li J., Li Y., Chen Z. (2018). Phenolic contents, cellular antioxidant activity and antiproliferative capacity of different varieties of oats. Food Chem..

[11] Kilci A., Gocmen D. (2014). Changes in antioxidant activity and phenolic acid composition of tarhana with steel-cut oats. Food Chem..

[12] Varga M., Jojart R., Fonad P., Mihaly R., Palagyi A. (2018). Phenolic composition and antioxidant activity of colored oats. Food Chem..

[13] Bei Q., Wu Z., Chen G. (2020). Dynamic changes in the phenolic composition and antioxidant activity of oats during simultaneous hydrolysis and fermentation. Food Chem..

[14] Kaminski K., Hac-Wydro K., Skora M., Tymecka M., Obloza M. (2022). Preliminary Studies on the Mechanism of Antifungal Activity of New Cationic beta-Glucan Derivatives Obtained from Oats and Barley. ACS Omega.

[15] Liu B., Lin Q., Yang T., Zeng L., Shi L., Chen Y., Luo F. (2015). Oat beta-glucan ameliorates dextran sulfate sodium (DSS)-induced ulcerative colitis in mice. Food Funct..

[16] Bai J., Zhao J., Al-Ansi W., Wang J., Xue L., Liu J., Wang Y., Fan M., Qian H., Li Y. (2021). Oat beta-glucan alleviates DSS-induced colitis via regulating gut microbiota metabolism in mice. Food Funct..

[17] Chudan S., Ishibashi R., Nishikawa M., Tabuchi Y., Nagai Y., Ikushiro S., Furusawa Y. (2023). Effect of soluble oat fiber on intestinal microenvironment and TNBS-induced colitis. Food Funct..

[18] Immonen M., Myllyviita J., Sontag-Strohm T., Myllarinen P. (2021). Oat Protein Concentrates with Improved Solubility Produced by an Enzyme-Aided Ultrafiltration Extraction Method. Foods.

[19] Sargautis D., Kince T. (2023). Effect of Enzymatic Pre-Treatment on Oat Flakes Protein Recovery and Properties. Foods.

[20] Wang H., Xie L., Liu S., Dai A., Chi X., Zhang D. (2022). Non-targeted metabolomics and microbial analyses of the impact of oat antimicrobial peptides on rats with dextran sulfate sodium-induced enteritis. Front. Nutr..

[21] Esfandi R., Willmore W.G., Tsopmo A. (2019). Peptidomic analysis of hydrolyzed oat bran proteins, and their in vitro antioxidant and metal chelating properties. Food Chem..

[22] Zhang J., Liang F., Chen Z., Chen Y., Yuan J., Xiong Q., Hou S., Huang S., Liu C., Liang J. (2022). Vitexin Protects against Dextran Sodium Sulfate-Induced Colitis in Mice and Its Potential Mechanisms. J. Agric. Food Chem..

[23] Yang W., Huang Z., Xiong H., Wang J., Zhang H., Guo F., Wang C., Sun Y. (2022). Rice Protein Peptides Alleviate Dextran Sulfate Sodium-Induced Colitis via the Keap1-Nrf2 Signaling Pathway and Regulating Gut Microbiota. J. Agric. Food Chem..

[24] Chen K., Gao C., Tang M., Dong Q., Wang N., Man S., Lu F., Wang H. (2022). Dietary soybeans worsen dextran sodium sulfate-induced colitis by disrupting intestinal ecology. Food Funct..

[25] Shimizu T., Hirano H., Shimizu S., Kishioka C., Sakakura Y., Majima Y. (2001). Differential properties of mucous glycoproteins in rat nasal epithelium. A comparison between allergic inflammation and lipopolysaccharide stimulation. Am. J. Respir. Crit. Care Med..

[26] Spatz M., Ciocan D., Merlen G., Rainteau D., Humbert L., Gomes-Rochette N., Hugot C., Trainel N., Mercier-Nome F., Domenichini S. (2021). Bile acid-receptor TGR5 deficiency worsens liver injury in alcohol-fed mice by inducing intestinal microbiota dysbiosis. JHEP Rep..

[27] Qi-Xiang M., Yang F., Ze-Hua H., Nuo-Ming Y., Rui-Long W., Bin-Qiang X., Jun-Jie F., Chun-Lan H., Yue Z. (2022). Intestinal TLR4 deletion exacerbates acute pancreatitis through gut microbiota dysbiosis and Paneth cells deficiency. Gut Microbes.

[28] Rajendhran J., Gunasekaran P. (2011). Microbial phylogeny and diversity: Small subunit ribosomal RNA sequence analysis and beyond. Microbiol. Res..

[29] Rudi K., Zimonja M., Trosvik P., Naes T. (2007). Use of multivariate statistics for 16S rRNA gene analysis of microbial communities. Int. J. Food Microbiol..

[30] Chang F., He S., Dang C. (2022). Assisted Selection of Biomarkers by Linear Discriminant Analysis Effect Size (LEfSe) in Microbiome Data. J. Vis. Exp..

[31] Zhang S., Wang H., Zhu M.J. (2019). A sensitive GC/MS detection method for analyzing microbial metabolites short chain fatty acids in fecal and serum samples. Talanta.

[32] Hsu Y.L., Chen C.C., Lin Y.T., Wu W.K., Chang L.C., Lai C.H., Wu M.S., Kuo C.H. (2019). Evaluation and Optimization of Sample Handling Methods for Quantification of Short-Chain Fatty Acids in Human Fecal Samples by GC-MS. J. Proteome Res..

[33] Recinella L., Gorica E., Chiavaroli A., Fraschetti C., Filippi A., Cesa S., Cairone F., Martelli A., Calderone V., Veschi S. (2022). Anti-Inflammatory and Antioxidant Effects Induced by Allium sativum L. Extracts on an Ex Vivo Experimental Model of Ulcerative Colitis. Foods.

[34] Peterson L.W., Artis D. (2014). Intestinal epithelial cells: Regulators of barrier function and immune homeostasis. Nat. Rev. Immunol..

[35] Mikocka-Walus A., Knowles S.R., Keefer L., Graff L. (2016). Controversies Revisited: A Systematic Review of the Comorbidity of Depression and Anxiety with Inflammatory Bowel Diseases. Inflamm. Bowel Dis..

[36] Neuendorf R., Harding A., Stello N., Hanes D., Wahbeh H. (2016). Depression and anxiety in patients with Inflammatory Bowel Disease: A systematic review. J. Psychosom. Res..

[37] Horst S., Chao A., Rosen M., Nohl A., Duley C., Wagnon J.H., Beaulieu D.B., Taylor W., Gaines L., Schwartz D.A. (2015). Treatment with immunosuppressive therapy may improve depressive symptoms in patients with inflammatory bowel disease. Dig. Dis. Sci..

[38] Kennedy D.O., Bonnlander B., Lang S.C., Pischel I., Forster J., Khan J., Jackson P.A., Wightman E.L. (2020). Acute and Chronic Effects of Green Oat (*Avena sativa*) Extract on Cognitive Function and Mood during a Laboratory Stressor in Healthy Adults: A Randomised, Double-Blind, Placebo-Controlled Study in Healthy Humans. Nutrients.

[39] Deleu S., Machiels K., Raes J., Verbeke K., Vermeire S. (2021). Short chain fatty acids and its producing organisms: An overlooked therapy for IBD?. EBioMedicine.

[40] Yamamoto M., Kensler T.W., Motohashi H. (2018). The KEAP1-NRF2 System: A Thiol-Based Sensor-Effector Apparatus for Maintaining Redox Homeostasis. Physiol. Rev..

[41] Bellezza I., Giambanco I., Minelli A., Donato R. (2018). Nrf2-Keap1 signaling in oxidative and reductive stress. Biochim. Biophys. Acta Mol. Cell Res..

[42] Lu M.C., Ji J.A., Jiang Z.Y., You Q.D. (2016). The Keap1-Nrf2-ARE Pathway as a Potential Preventive and Therapeutic Target: An Update. Med. Res. Rev..

[43] Tontini G.E., Vecchi M., Pastorelli L., Neurath M.F., Neumann H. (2015). Differential diagnosis in inflammatory bowel disease colitis: State of the art and future perspectives. World J. Gastroenterol..

[44] Ng S.C., Shi H.Y., Hamidi N., Underwood F.E., Tang W., Benchimol E.I., Panaccione R., Ghosh S., Wu J.C.Y., Chan F.K.L. (2017). Worldwide incidence and prevalence of inflammatory bowel disease in the 21st century: A systematic review of population-based studies. Lancet.

[45] Rogler G. (2014). Chronic ulcerative colitis and colorectal cancer. Cancer Lett..

[46] Zhang B., Xu Y., Zhao C., Zhang Y., Lv H., Ji X., Wang J., Pang W., Wang X., Wang S. (2022). Protective effects of bioactive peptides in foxtail millet protein hydrolysates against experimental colitis in mice. Food Funct..

[47] Mazur M., Wlodarczyk J., Swierczynski M., Kordek R., Grzybowski M.M., Olczak J., Fichna J. (2022). The Anti-Inflammatory Effect of Acidic Mammalian Chitinase Inhibitor OAT-177 in DSS-Induced Mouse Model of Colitis. Int. J. Mol. Sci..

[48] Huang Z., Yang W., Wang X., Guo F., Cheng Y., Cao L., Zhu W., Sun Y., Xiong H. (2022). Industrially Produced Rice Protein Ameliorates Dextran Sulfate Sodium-Induced Colitis via Protecting the Intestinal Barrier, Mitigating Oxidative Stress, and Regulating Gut Microbiota. J. Agric. Food Chem..

[49] Wang F., Chen Y., Itagaki K., Zhu B., Lin Y., Song H., Wang L., Xiong L., Weng Z., Shen X. (2023). Wheat Germ-Derived Peptide Alleviates Dextran Sulfate Sodium-Induced Colitis in Mice. J. Agric. Food Chem..

[50] Hong Z., Shi C., Hu X., Chen J., Li T., Zhang L., Bai Y., Dai J., Sheng J., Xie J. (2023). Walnut Protein Peptides Ameliorate DSS-Induced Ulcerative Colitis Damage in Mice: An In Silico Analysis and in Vivo Investigation. J. Agric. Food Chem..

[51] Li J.W., Fang B., Pang G.F., Zhang M., Ren F.Z. (2019). Age- and diet-specific effects of chronic exposure to chlorpyrifos on hormones, inflammation and gut microbiota in rats. Pestic. Biochem. Physiol..

[52] He B., Li T., Wang W., Gao H., Bai Y., Zhang S., Zang J., Li D., Wang J. (2019). Metabolic characteristics and nutrient utilization in high-feed-efficiency pigs selected using different feed conversion ratio models. Sci. China Life Sci..

[53] Pittayanon R., Lau J.T., Leontiadis G.I., Tse F., Yuan Y., Surette M., Moayyedi P. (2020). Differences in Gut Microbiota in Patients with vs without Inflammatory Bowel Diseases: A Systematic Review. Gastroenterology.

[54] Lv L.X., Li Y.D., Hu X.J., Shi H.Y., Li L.J. (2014). Whole-genome sequence assembly of Pediococcus pentosaceus LI05 (CGMCC 7049) from the human gastrointestinal tract and comparative analysis with representative sequences from three food-borne strains. Gut Pathog..

[55] Dong F., Xiao F., Li X., Li Y., Wang X., Yu G., Zhang T., Wang Y. (2022). Pediococcus pentosaceus CECT 8330 protects DSS-induced colitis and regulates the intestinal microbiota and immune responses in mice. J. Transl. Med..

[56] Bian X., Yang L., Wu W., Lv L., Jiang X., Wang Q., Wu J., Li Y., Ye J., Fang D. (2020). Pediococcus pentosaceus LI05 alleviates DSS-induced colitis by modulating immunological profiles, the gut microbiota and short-chain fatty acid levels in a mouse model. Microb. Biotechnol..

[57] Quaglio A.E.V., Grillo T.G., De Oliveira E.C.S., Di Stasi L.C., Sassaki L.Y. (2022). Gut microbiota, inflammatory bowel disease and colorectal cancer. World J. Gastroenterol..

[58] Cao Y., Wang Z., Yan Y., Ji L., He J., Xuan B., Shen C., Ma Y., Jiang S., Ma D. (2021). Enterotoxigenic Bacteroidesfragilis Promotes Intestinal Inflammation and Malignancy by Inhibiting Exosome-Packaged miR-149-3p. Gastroenterology.

[59] Lavelle A., Sokol H. (2020). Gut microbiota-derived metabolites as key actors in inflammatory bowel disease. Nat. Rev. Gastroenterol. Hepatol..

[60] Banfi D., Moro E., Bosi A., Bistoletti M., Cerantola S., Crema F., Maggi F., Giron M.C., Giaroni C., Baj A. (2021). Impact of Microbial Metabolites on Microbiota-Gut-Brain Axis in Inflammatory Bowel Disease. Int. J. Mol. Sci..

[61] Ott C., Scholmerich J. (2013). Extraintestinal manifestations and complications in IBD. Nat. Rev. Gastroenterol. Hepatol..

[62] Ferro J.M., Oliveira Santos M. (2021). Neurology of inflammatory bowel disease. J. Neurol. Sci..

[63] Zamani M., Alizadeh-Tabari S., Singh S., Loomba R. (2022). Meta-analysis: Prevalence of, and risk factors for, non-alcoholic fatty liver disease in patients with inflammatory bowel disease. Aliment. Pharmacol. Ther..

