# Supplementary material for: Oat Peptides Alleviate Dextran Sulfate Sodium Salt-Induced Colitis by Maintaining the Intestinal Barrier and Modulating the Keap1-Nrf2 Axis
Source: Nutrients. 2023 Dec 9;15(24):5055. doi: 10.3390/nu15245055 (PMC10746067; doi:10.3390/nu15245055)
Supplement: Supplementary file 1 [file nutrients-15-05055-s001.zip › nutrients-2749494-supplementary.docx]

Oat peptides alleviate DSS-induced colitis by maintaining the intestinal barrier and modulating the Keap1-Nrf2 axis

Zhong-Hao Ji^1,2 †^, Wen-Yin Xie^1 †^, Pei-Sen Zhao^1^, Hong-Yu Wu^1,3^, Wen-Zhi Ren^1^, Jin-Ping Hu^1^, Bao Yuan1 and Wei Gao1*

1 Department of Laboratory Animals, College of Animal Sciences, Jilin University, Changchun 130062, Jilin, China

2 Department of Basic Medicine, Changzhi Medical College, Changzhi 046000, Shanxi, China

3 Jilin Academy of Agricultural Sciences, Jilin 132101, China

* Corresponding author

Tel.: +86-0431-87836536

1. mail address: [gaowei81@jlu.edu.cn](mailto:gaowei81@jlu.edu.cn)


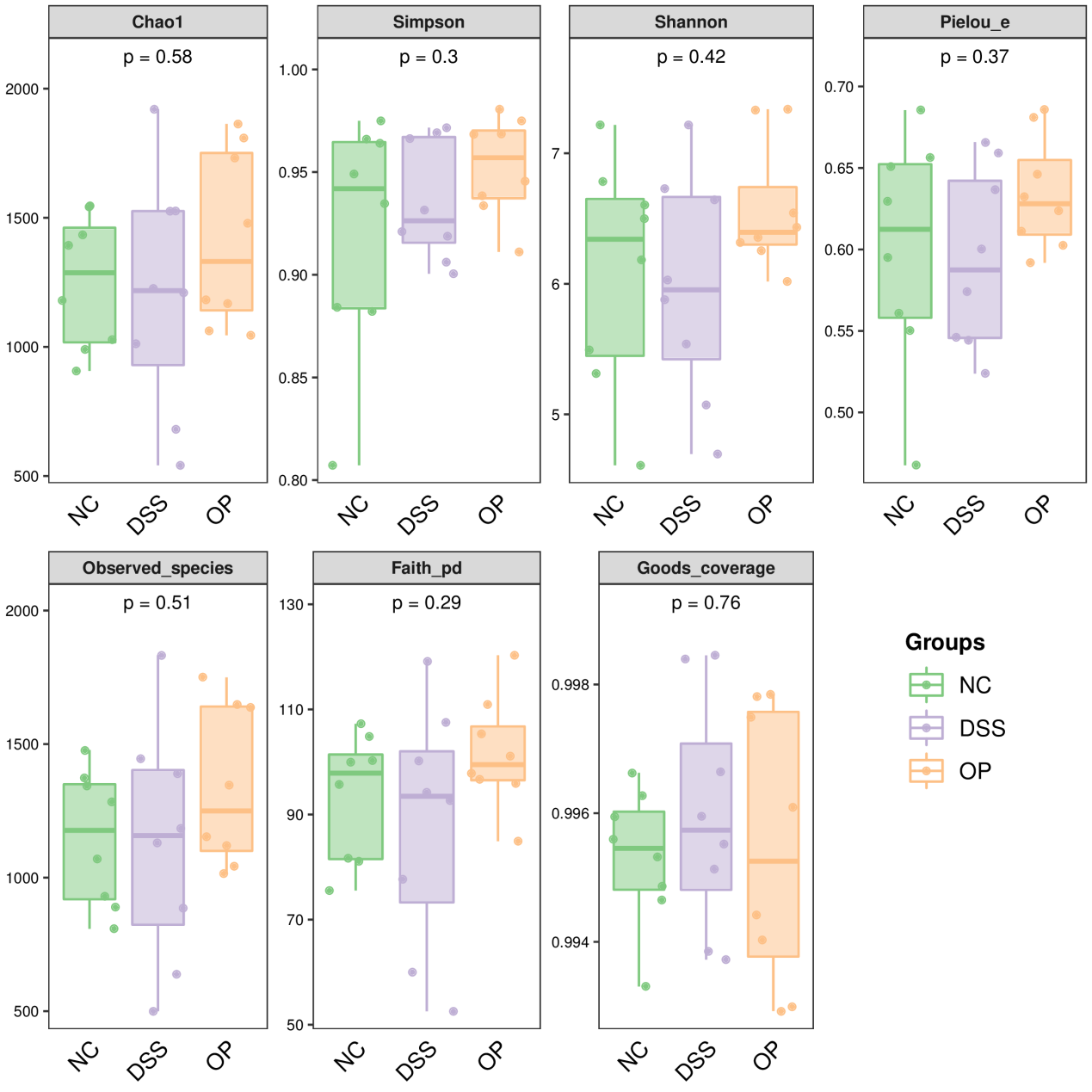


Figure S1. Results of alpha diversity analysis


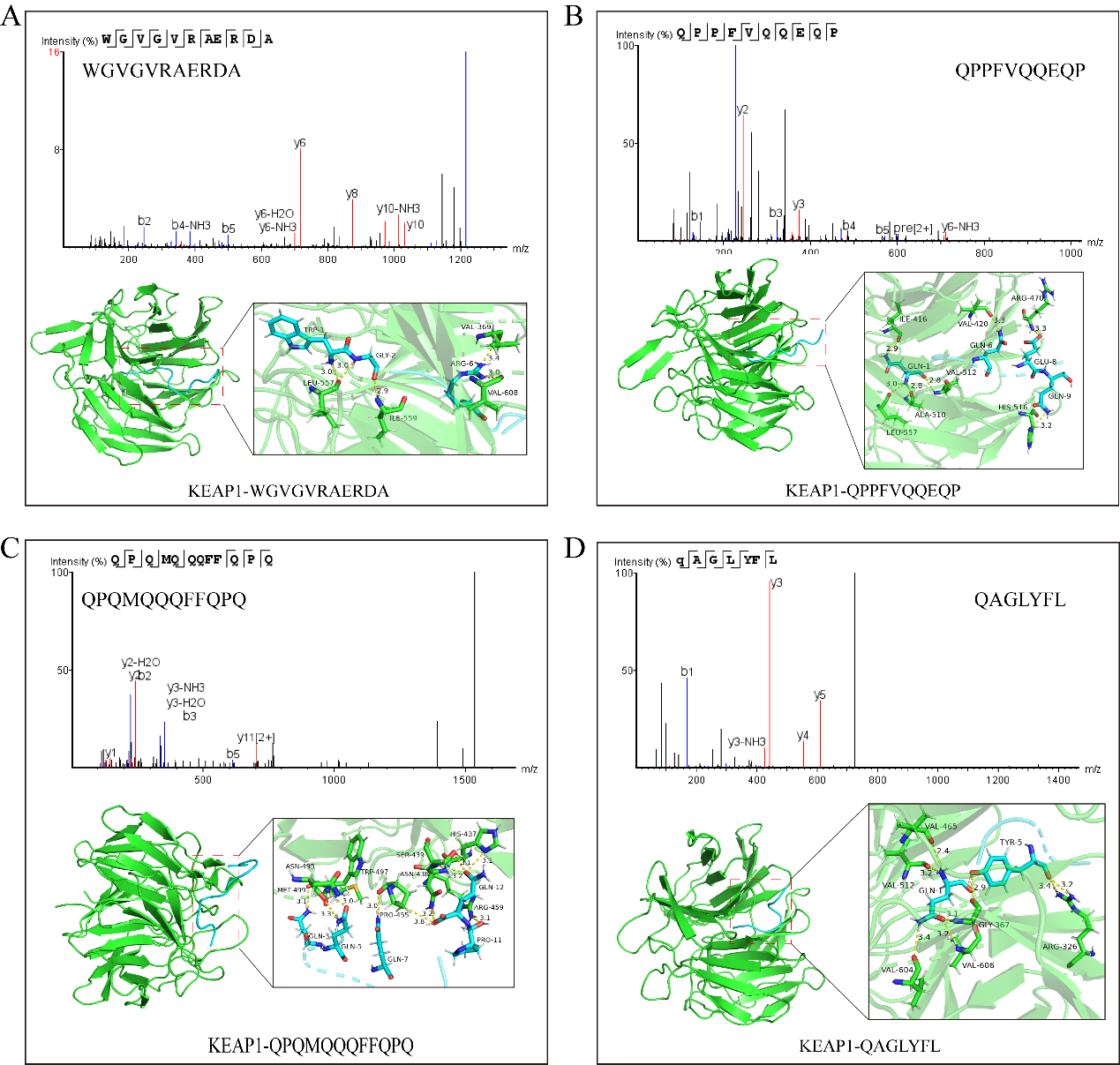


Figure S2. Molecular docking prediction of the interactions of four bioactive peptides with the receptor protein Keap1. A-D: Mass spectrometry identification results for four peptides, WGVGVRAERDA, QPPFVQQEQP, QPQMQQQFFQPQ and QAGLYFL, and the molecular docking results for the binding of these peptides with the receptor protein Keap1. In the molecular docking diagram, the blue structure is the ligand peptide, and the green structure is the receptor Keap1 protein.


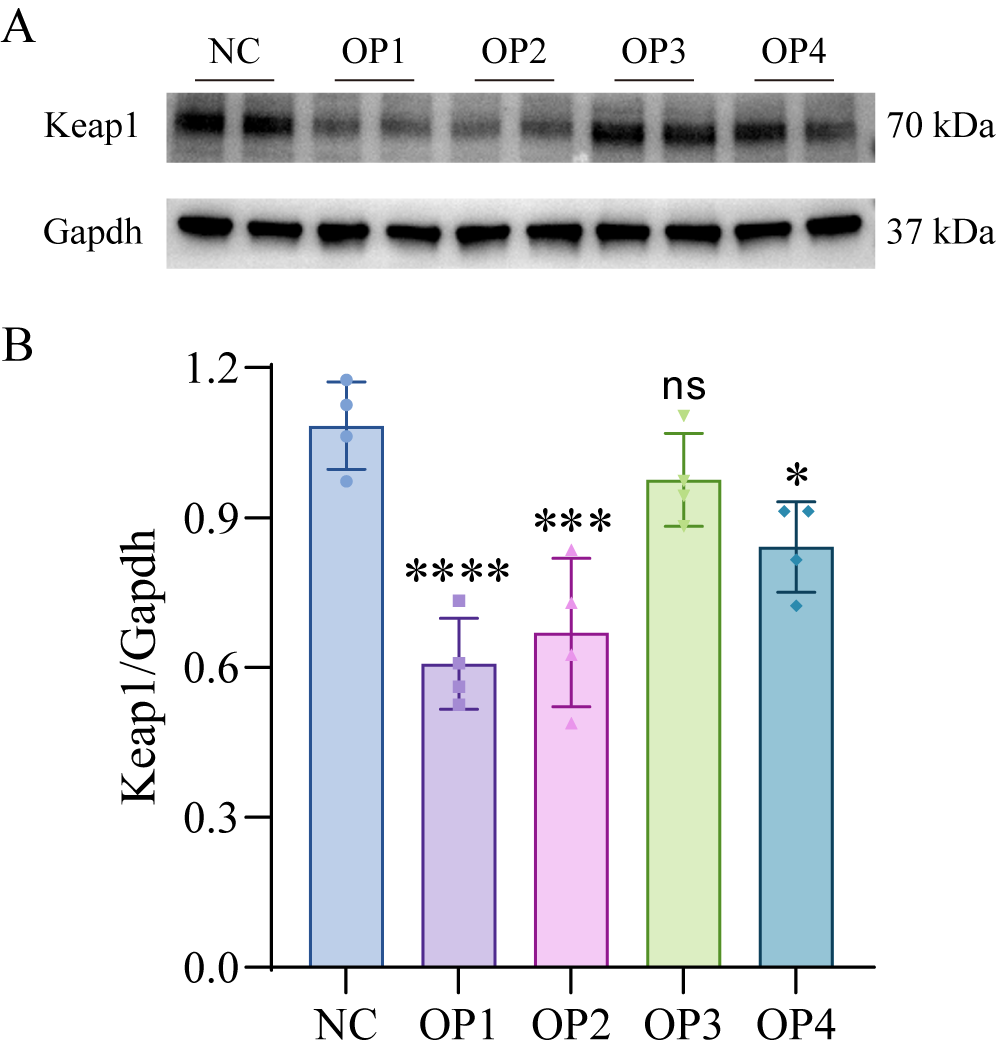


Figure S3. WB analysis of the effect of four peptides on Keap1. A: representative WB results; B: quantitative analysis results. WGVGVRAERDA (OP1), QPPFVQQEQP (OP2), QPQMQQQQFFQPQ (OP3) and QAGLYFL (OP4). * *P* < 0.05, *** *P* < 0.001, **** *P* < 0.0001, ns *P* > 0.05


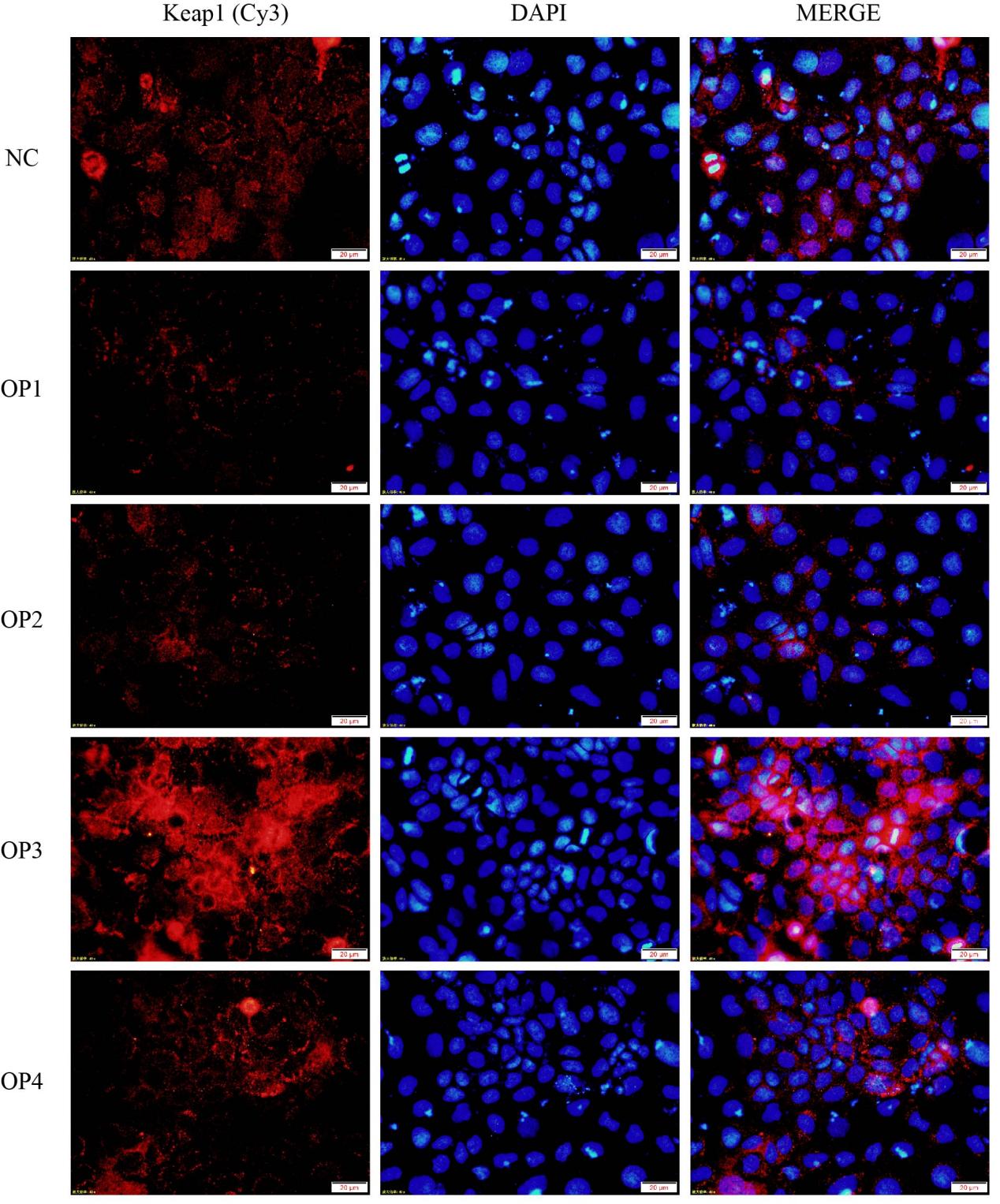


Figure S4. IF analysis of the effect of four peptides on Keap1. WGVGVRAERDA (OP1), QPPFVQQEQP (OP2), QPQMQQQQFFQPQ (OP3) and QAGLYFL (OP4).


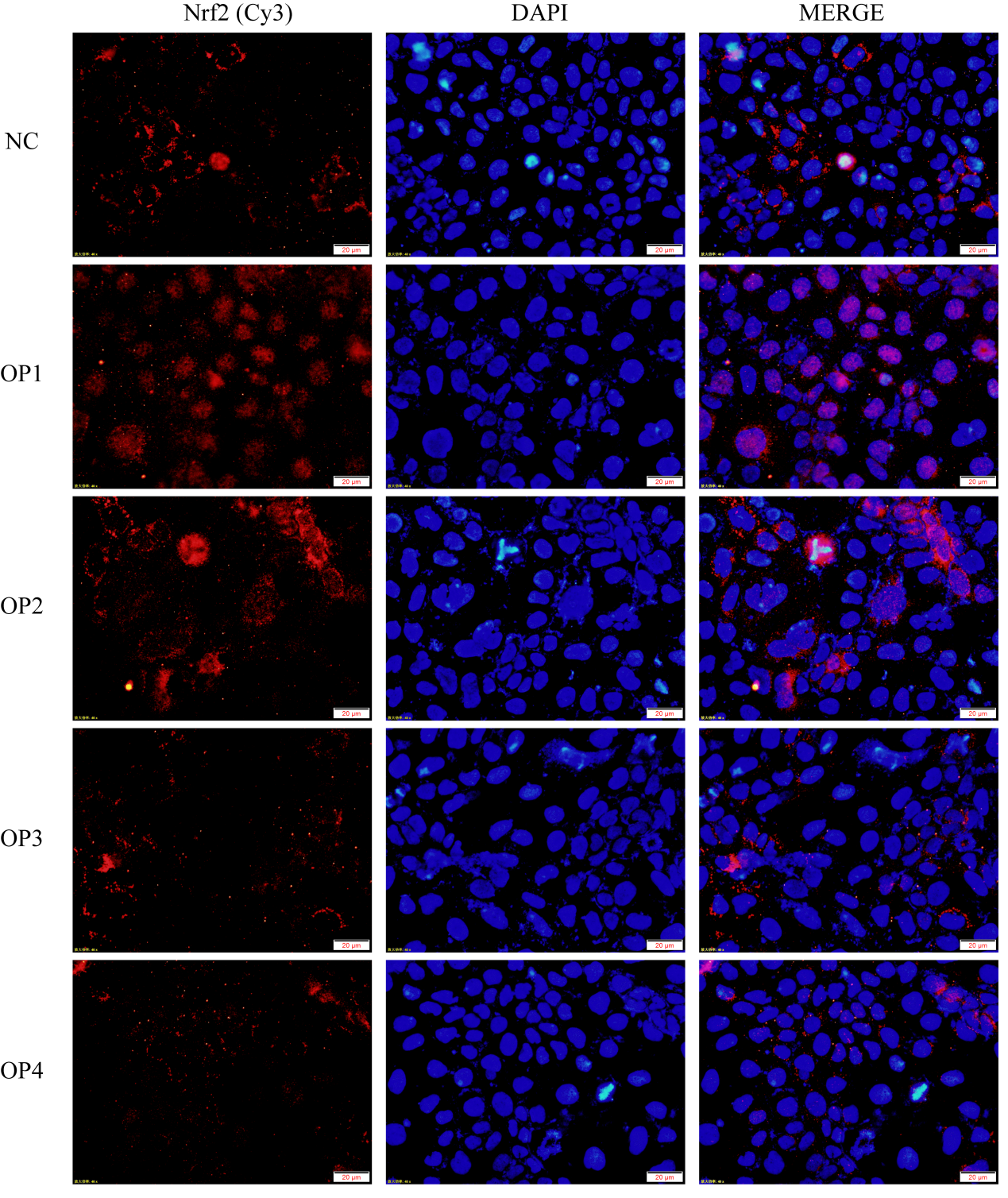


Figure S5. IF analysis of the effect of four peptides on Nrf2. WGVGVRAERDA (OP1), QPPFVQQEQP (OP2), QPQMQQQQFFQPQ (OP3) and QAGLYFL (OP4).

Table S1. The assessment criteria for DAI scoring

| score | weight loss(%) | fecal trait | fecal blood |
| --- | --- | --- | --- |
| 0 | weight does not change | normal | normal |
| 1 | weight loss≤5% | soft but still shaped |  |
| 2 | 5< weight loss ≤ 10% | loose stool | hemoccult positive |
| 3 | 10< weight loss ≤ 15% | very soft |  |
| 4 | 15% < weight loss | diarrhea | severe bleeding |

Table S2. Primers of RT-qPCR

| NCBI ID | Primer name | Sequence (5 '-3') | Product size (bp) |
| --- | --- | --- | --- |
| *m-IL-1β* | Forward primer | TGGACCTTCCAGGATGAGGACA | 148 |
| NM_008361 | Reverse primer | GTTCATCTCGGAGCCTGTAGTG |  |
| *m-IL-6* | Forward primer | CTGCAAGAGACTTCCATCCAG | 131 |
| NM_031168 | Reverse primer | AGTGGTATAGACAGGTCTGTTGG |  |
| *m-TNF-α* | Forward primer | GGTGCCTATGTCTCAGCCTCTT | 139 |
| NM_013693 | Reverse primer | GCCATAGAACTGATGAGAGGGAG |  |
| *m-Cldn1*（claudin 1） | Forward primer | GGGGACAACATCGTGACCG | 100 |
| NM_016674.4 | Reverse primer | AGGAGTCGAAGACTTTGCACT |  |
| *m-Tjp1*（ZO-1） | Forward primer | GCCGCTAAGAGCACAGCAA | 134 |
| NM_009386.2 | Reverse primer | TCCCCACTCTGAAAATGAGGA |  |
| *m-Ocln*（occludin） | Forward primer | TTGAAAGTCCACCTCCTTACAGA | 129 |
| NM_008756.2 | Reverse primer | CCGGATAAAAAGAGTACGCTGG |  |
| *m-Actb* | Forward primer | GGCTGTATTCCCCTCCATCG | 154 |
| NM_007393.5 | Reverse primer | CCAGTTGGTAACAATGCCATGT |  |
| *h-NQO1* | Forward primer | ATTGGACCGAGCTGGAA | 134 |
| NM_000903.3 | Reverse primer | GCAGTGAAGATGAAGGCAA |  |
| *h-HO-1* | Forward primer | GTGACCCGAGACGGCTT | 107 |
| NM_002133.3 | Reverse primer | ACAGGGGCGAAGACTGG |  |
| *h-ACTB* | Forward primer | CTTCGCGGGCGACGAT | 104 |
| NM_001101.5 | *Reverse primer* | *CCACATAGGAATCCTTCTGACC* |  |

Table S3. Amino acid composition of OPs

| Amino acid | content (g per 100g) |
| --- | --- |
| Asp | 5.3288 |
| Thr | 1.8569 |
| Ser | 3.4026 |
| Glu | 15.7988 |
| Pro | 12.9541 |
| Gly | 19.9101 |
| Ala | 8.2546 |
| Cys | NA |
| Val | 2.5872 |
| Met | 0.5256 |
| Ile | 1.7899 |
| Leu | 3.4958 |
| Tyr | 0.8954 |
| Phe | 2.2114 |
| Lys | 3.6896 |
| His | 0.9150 |
| Arg | 6.7705 |
| Total | 90.3863 |

*NA means not detected

Table S4. Composition of peptides in OPs

| Peptide | -10lgP | Mass (Da) | Length | Error (ppm) | Experimental m/z | Retention time（min） | Area Sample 1 | Accession | Peptide Ranker score | percentage of OPs(%) |
| --- | --- | --- | --- | --- | --- | --- | --- | --- | --- | --- |
| WGVGVRAERDA | 19.81 | 1214.6156 | 11 | -3.2 | 608.3167 | 21.46 | 8.59E+08 |  | 0.151609 | 33.55% |
| QPPFVQQEQP | 20.85 | 1196.5825 | 10 | 6 | 599.3057 | 96.73 | 4.66E+08 |  | 0.21875 | 18.19% |
| QPQMQQQFFQPQ | 24.69 | 1533.7034 | 12 | 7.5 | 767.8693 | 86.7 | 1.36E+08 |  | 0.363422 | 5.33% |
| STPAPAPAPA | 22.09 | 878.4497 | 10 | -0.2 | 440.2346 | 24.19 | 1.04E+08 |  | 0.535619 | 4.05% |
| G(+42.01)LVQPQTQMAGQVFIQPQQLAQYQAM(+15.99)KVVAM(+15.99)QT | 17.3 | 3733.863 | 33 | 0.3 | 934.4788 | 27.07 | 9.46E+07 |  | 0.0228891 | 3.70% |
| Q(+42.01)PQLQQVFNQPQ | 25.94 | 1495.7419 | 12 | -7.8 | 748.8768 | 72.63 | 9.34E+07 |  | 0.210345 | 3.65% |
| QMGLVQPQTQMASQVFIQPQQLPQYQA | 22.01 | 3086.5317 | 27 | 3.3 | 618.3193 | 20.21 | 7.07E+07 |  | 0.108174 | 2.76% |
| QPPFVQQEQPFVQQQ | 15.86 | 1826.8951 | 15 | -6.4 | 914.4544 | 43.21 | 7.02E+07 |  | 0.191547 | 2.74% |
| Q(+42.01)AGLYFL | 16.43 | 852.4381 | 7 | 6.6 | 427.2317 | 12.02 | 5.89E+07 |  | 0.842295 | 2.30% |
| EFPLGYKTFGEAIPPQ | 22.27 | 1792.9036 | 16 | -8.4 | 897.4568 | 104.62 | 5.55E+07 |  | 0.478492 | 2.17% |
| Q(+42.01)VSQPQLQLQQQVFQPQ | 18.3 | 2065.0593 | 17 | 9.6 | 1033.553 | 57.25 | 4.56E+07 |  | 0.150762 | 1.78% |
| PQLQQVFNQPQ | 20.52 | 1325.6727 | 11 | 4 | 663.8502 | 78.7 | 3.91E+07 |  | 0.185726 | 1.53% |
| E(+42.01)QQQSILQQQQM(+15.99)LLQQQQQM(+15.99)LL | 16.08 | 2785.3738 | 22 | -2.3 | 697.3533 | 35.86 | 3.29E+07 |  | 0.136388 | 1.28% |
| A(+42.01)GERPEEAAVQPQ | 23.59 | 1422.6738 | 13 | 7.9 | 712.354 | 35.11 | 2.57E+07 |  | 0.103102 | 1.00% |
| HYINNSQALRSGI | 24.12 | 1471.7532 | 13 | 1.7 | 368.9484 | 17.66 | 2.21E+07 |  | 0.166901 | 0.86% |
| RVIPLVD | 20.13 | 810.4963 | 7 | -0.2 | 406.2578 | 43.06 | 2.20E+07 |  | 0.119074 | 0.86% |
| QQPPFVQQEQP | 18.07 | 1324.6411 | 11 | 4.3 | 663.3346 | 94.14 | 2.10E+07 |  | 0.230543 | 0.82% |
| QQQPFLQQQPLLQQQQ | 15.14 | 1979.0225 | 16 | 5.9 | 660.6892 | 61.11 | 2.00E+07 |  | 0.276478 | 0.78% |
| NKSDAKFLVQPGMALDNTPSAGEW | 15.09 | 2575.2375 | 24 | 9.7 | 644.8267 | 9.96 | 1.90E+07 |  | 0.113992 | 0.74% |
| G(+42.01)LAEGLLLEPPPQ | 16.69 | 1374.7394 | 13 | -8.8 | 688.375 | 68.45 | 1.83E+07 |  | 0.293526 | 0.72% |
| LRC(+57.02)PAIHSVVQAIIMQQQQFFQPQ | 15.78 | 2866.4734 | 24 | 5.6 | 717.6339 | 25.84 | 1.77E+07 |  | 0.152291 | 0.69% |
| EPFVQQQPPFVQ | 18.74 | 1442.7194 | 12 | 0.9 | 722.3719 | 75.94 | 1.40E+07 |  | 0.324557 | 0.55% |
| IDLPGC(+57.02)PR | 33.51 | 926.4644 | 8 | 3.3 | 464.2437 | 33.85 | 1.33E+07 | A0A1B2LQE8:A0A1B2LQC6:A0A1B2LQD6 | 0.74908 | 0.52% |
| ATPVAGAGTA | 22.43 | 814.4185 | 10 | 7.1 | 408.2218 | 9.25 | 1.15E+07 |  | 0.167768 | 0.45% |
| QPQM(+15.99)QQVTQGIFLPQ | 20.45 | 1757.8771 | 15 | 7.2 | 879.9573 | 104.73 | 8.96E+06 |  | 0.287778 | 0.35% |
| QPQAQM(+15.99)AGQVFIQPQ | 19.24 | 1685.8195 | 15 | 2.8 | 843.9243 | 96.31 | 8.79E+06 |  | 0.321288 | 0.34% |
| QQPPFVQQEQPFVQQQPFVQQEQP | 20.1 | 2908.4143 | 24 | 4.5 | 970.4888 | 99.89 | 8.55E+06 |  | 0.194618 | 0.33% |
| RATVDGAR | 18.68 | 844.4515 | 8 | -1.5 | 423.2349 | 17.15 | 7.79E+06 |  | 0.137288 | 0.30% |
| FFAAFVVA | 24.78 | 870.4639 | 8 | -9.5 | 436.2377 | 21.27 | 7.68E+06 |  | 0.534515 | 0.30% |
| K(+42.01)GEIGKH | 19.79 | 809.4395 | 7 | -1.7 | 405.7287 | 12.23 | 6.49E+06 |  | 0.134991 | 0.25% |
| LSRLSPR | 19.42 | 827.4977 | 7 | -3.9 | 414.757 | 10.08 | 5.93E+06 |  | 0.335717 | 0.23% |
| K(+42.01)PGGLATLREMYDEWPF | 20.25 | 2050.9822 | 17 | -5.4 | 411.2039 | 17.06 | 5.88E+06 |  | 0.632267 | 0.23% |
| KDGDGAERNEC(+57.02) | 17.19 | 1249.4993 | 11 | 1.5 | 625.7615 | 99.1 | 5.85E+06 |  | 0.245205 | 0.23% |
| GLIDLPGC(+57.02)PR | 42 | 1096.5698 | 10 | 7.3 | 549.2994 | 58.09 | 5.73E+06 | A0A1B2LQE8:A0A1B2LQC6:A0A1B2LQD6 | 0.836335 | 0.22% |
| GSSC(+57.02)RSVDRL | 21.67 | 1135.5404 | 10 | 6.6 | 568.7846 | 38.41 | 5.47E+06 |  | 0.572581 | 0.21% |
| QPQLLAQLQQGPQ | 16.91 | 1447.7783 | 13 | 1.2 | 724.9016 | 92.94 | 5.19E+06 |  | 0.299783 | 0.20% |
| AIWTSIQGDLSGFK | 45.28 | 1521.7827 | 14 | -4.6 | 761.8997 | 96.66 | 5.06E+06 | I0J8Q6 | 0.456791 | 0.20% |
| TAFVQFPQ | 19.13 | 936.4705 | 8 | 2.8 | 469.2466 | 88.25 | 4.95E+06 |  | 0.346716 | 0.19% |
| S(+42.01)REPVVKI | 15.97 | 968.5654 | 8 | 6 | 323.8663 | 23.69 | 4.92E+06 |  | 0.138621 | 0.19% |
| TKPNSSSRQKAQKPSPDAGARPPSSGAQ | 18.06 | 2834.4382 | 28 | 8.7 | 567.9032 | 42.71 | 4.63E+06 |  | 0.215106 | 0.18% |
| QPFVQQQQPFVQQQQMFLQP | 19.12 | 2473.2212 | 20 | -6.7 | 619.3121 | 75.6 | 4.50E+06 |  | 0.335091 | 0.18% |
| LMADPQGQM(+15.99)IDLPI | 15.87 | 1556.7578 | 14 | 2.7 | 779.3929 | 108.17 | 4.24E+06 |  | 0.320061 | 0.17% |
| QPQLQQEVFQP | 20.89 | 1340.6725 | 11 | -6.3 | 671.3433 | 103.88 | 4.01E+06 |  | 0.222757 | 0.16% |
| KDPYADLYVVRWTP | 17.11 | 1721.8777 | 14 | -1.4 | 861.95 | 80.37 | 3.70E+06 |  | 0.556885 | 0.14% |
| QQPFMQQKQPFM(+15.99)QQQQQP | 18.43 | 2290.0623 | 18 | 4 | 1146.0498 | 99.12 | 3.69E+06 |  | 0.231458 | 0.14% |
| QQPFM(+15.99)QQKQPFMQQQQQP | 15.94 | 2290.0623 | 18 | 4 | 1146.0498 | 99.12 | 3.69E+06 |  | 0.231458 | 0.14% |
| KGDAAVLPPEFSA | 17.99 | 1300.6663 | 13 | 2.1 | 434.5662 | 8.43 | 3.48E+06 |  | 0.47776 | 0.14% |
| Q(+42.01)QLSQYQTMKV | 16.78 | 1394.6864 | 11 | -6 | 698.3504 | 36.84 | 3.37E+06 |  | 0.236304 | 0.13% |
| QPQLQQQVFQPQ | 21.24 | 1467.7469 | 12 | -0.9 | 734.8844 | 105.22 | 3.16E+06 |  | 0.202432 | 0.12% |
| Q(+42.01)FLNERLA | 16.07 | 1031.5399 | 8 | 5.6 | 516.7832 | 57.69 | 3.13E+06 |  | 0.287122 | 0.12% |
| PQLQLQQQVFQPQ | 19.3 | 1580.8311 | 13 | -2.2 | 791.4258 | 88.98 | 3.03E+06 |  | 0.173636 | 0.12% |
| VIDAPGHRDFIK | 38.01 | 1366.7357 | 12 | -5.3 | 342.6914 | 24.32 | 2.92E+06 | A0A411EWN3 | 0.460275 | 0.11% |
| SVRPEDPHIQP | 21.16 | 1273.6415 | 11 | -0.9 | 637.8312 | 30.6 | 2.92E+06 |  | 0.257187 | 0.11% |
| PQLQQEVFQPQ | 28.18 | 1340.6725 | 11 | 5 | 671.3508 | 92.27 | 2.84E+06 |  | 0.151354 | 0.11% |
| GVPVRGAAGP | 15.25 | 879.4926 | 10 | -3.2 | 440.7548 | 20.54 | 2.73E+06 |  | 0.346348 | 0.11% |
| Q(+42.01)VQQQLQQQLIQPQ | 15.99 | 1747.9216 | 14 | -3.6 | 874.9702 | 71.56 | 2.71E+06 |  | 0.0977004 | 0.11% |
| C(+57.02)NIIQGSIQGDLGGIFGFQR | 56.28 | 2179.0845 | 20 | -1 | 727.3724 | 104.8 | 2.56E+06 | J7FQW2:Q9M4E2:J7FKU9:J7FQW7:J7FK20 | 0.72587 | 0.10% |
| QQPPFVQQQQPFVQ | 17.2 | 1697.8525 | 14 | -1.8 | 849.937 | 100.32 | 2.40E+06 |  | 0.340411 | 0.09% |
| QEVFQPQ | 17.97 | 874.4185 | 7 | 9.5 | 438.2233 | 11.7 | 2.40E+06 |  | 0.169209 | 0.09% |
| PVVGVPQ | 16 | 694.4014 | 7 | 9.8 | 348.2134 | 11.85 | 2.30E+06 |  | 0.123915 | 0.09% |
| AASSPFDRQQGEARGTVAPPLPLPASSAGNDGQPQPS | 23.44 | 3657.7771 | 37 | 5.4 | 915.4619 | 23.04 | 2.11E+06 |  | 0.551704 | 0.08% |
| Q(+42.01)FLGNPVT | 18.56 | 916.4654 | 8 | 6.2 | 459.2455 | 78.59 | 1.98E+06 |  | 0.318398 | 0.08% |
| QQQPPFVQQEQP | 22.35 | 1452.6997 | 12 | -5.4 | 727.3575 | 86.65 | 1.88E+06 |  | 0.209137 | 0.07% |
| AQIPRQL | 19.32 | 824.4868 | 7 | -2.3 | 413.2522 | 26.9 | 1.85E+06 |  | 0.428662 | 0.07% |
| Q(+42.01)GDVIALPAG | 15.17 | 981.5131 | 10 | -4.6 | 491.7645 | 42.35 | 1.78E+06 |  | 0.467771 | 0.07% |
| C(+57.02)VRFC(+57.02)NKGAVNPSVG | 15.19 | 1663.7922 | 15 | -7.9 | 832.9017 | 8.56 | 1.73E+06 |  | 0.573431 | 0.07% |
| PGVVFDVDYLVRQEQFRFFTEAL | 16.67 | 2774.4067 | 23 | 3 | 555.8936 | 24.4 | 1.73E+06 |  | 0.0373852 | 0.07% |
| AARC(+57.02)VGSPLAA | 15.21 | 1071.5494 | 11 | -8 | 536.7809 | 32.26 | 1.63E+06 |  | 0.322451 | 0.06% |
| QPAPAM(+15.99)LSSAPQ | 16.24 | 1212.5808 | 12 | 9.9 | 607.3073 | 89.79 | 1.55E+06 |  | 0.412106 | 0.06% |
| QPQLQQQVFQLQLQQQAQVQQQVFQPQ | 15.05 | 3262.6846 | 27 | -4.1 | 653.5454 | 50.64 | 1.52E+06 |  | 0.117145 | 0.06% |
| TEAVDAITTHPGI | 17.03 | 1323.667 | 13 | 0.7 | 442.2325 | 34.14 | 1.30E+06 |  | 0.114242 | 0.05% |
| G(+42.01)LFHLSVRPPQ | 16.54 | 1291.7036 | 11 | -7.9 | 646.8578 | 96.96 | 1.28E+06 |  | 0.46771 | 0.05% |
| SWKGNIPQ | 15.81 | 928.4766 | 8 | 5.8 | 465.251 | 99.88 | 1.26E+06 |  | 0.433338 | 0.05% |
| LPPGFQERTRGQGMVTTGWLPQ | 15 | 2455.2429 | 22 | -6.6 | 819.421 | 108.22 | 1.17E+06 |  | 0.240018 | 0.05% |
| VPSRMHLEPQ | 20.85 | 1192.6023 | 10 | -5.9 | 597.3084 | 68.09 | 1.12E+06 |  | 0.183659 | 0.04% |
| QPQM(+15.99)QQVTQGIFQPQ | 15.21 | 1772.8516 | 15 | 0.5 | 887.4387 | 94.59 | 1.11E+06 |  | 0.149047 | 0.04% |
| SILGQC(+57.02)C(+57.02)EM(+15.99)PVGYVQLPVGVAGP | 17.12 | 2446.1694 | 23 | 5.7 | 612.5568 | 68.9 | 1.11E+06 |  | 0.738116 | 0.04% |
| RSPQDKNLYFEIQKQNLFASEMR | 22.19 | 2841.4231 | 23 | -2.5 | 711.3655 | 37.6 | 1.10E+06 |  | 0.053487 | 0.04% |
| GFQRDRASKVIQ | 15.98 | 1403.7633 | 12 | 1 | 468.9316 | 12.75 | 1.07E+06 | J7FQW2:Q9M4E2:J7FKU9:J7FQW7:J7FK20 | 0.184365 | 0.04% |
| G(+42.01)IMYPGFFQKTIWFFMDPLMHYVRY | 17.04 | 3228.5427 | 25 | -8.1 | 808.1412 | 22.06 | 1.05E+06 |  | 0.252797 | 0.04% |
| V(+42.01)C(+57.02)C(+57.02)SRLGQMPPQ | 15.07 | 1473.6527 | 12 | 2.2 | 737.8396 | 104.32 | 9.98E+05 | J7FQW2:Q9M4E2 | 0.448019 | 0.04% |
| LPKFKIS | 30.58 | 831.5218 | 7 | 1.3 | 416.7712 | 33.81 | 9.75E+05 |  | 0.318186 | 0.04% |
| KDFPVTW | 34.9 | 891.449 | 7 | 0.1 | 446.7345 | 59.69 | 9.55E+05 | J7FQW2:Q9M4E2:J7FKU9:J7FQW7:J7FK20 | 0.732923 | 0.04% |
| GPRVLGGGM(+15.99)IELHSWIPR | 15.21 | 1990.057 | 18 | -1.9 | 498.5235 | 10.08 | 8.62E+05 |  | 0.803743 | 0.03% |
| NYLLDRC(+57.02)SAMKDFPVTW | 15.21 | 2114.9917 | 17 | 4.1 | 529.7605 | 8.09 | 8.59E+05 | J7FKU9 | 0.595106 | 0.03% |
| AVSLPGGR | 15.47 | 755.429 | 8 | -4.3 | 378.7224 | 23.45 | 7.86E+05 |  | 0.610324 | 0.03% |
| QPIMQQQQLLLQQQQ | 16.07 | 1850.9673 | 15 | -0.2 | 926.4962 | 88.3 | 7.30E+05 |  | 0.121189 | 0.03% |
| Q(+42.01)QQPFVQQQQMFLQP | 24.88 | 1915.925 | 15 | -3.9 | 639.6503 | 16.45 | 7.18E+05 |  | 0.422457 | 0.03% |
| QLKDTGC(+57.02)GISPQ | 17.91 | 1302.6238 | 12 | -0.2 | 652.3229 | 94 | 6.86E+05 |  | 0.19399 | 0.03% |
| IGPLNPL | 15.47 | 722.4326 | 7 | -1.4 | 362.2252 | 71.22 | 6.64E+05 |  | 0.730193 | 0.03% |
| Q(+42.01)LQQEVFQPQLQQV | 15.2 | 1753.8999 | 14 | -3.3 | 585.6421 | 41.14 | 6.41E+05 |  | 0.129144 | 0.03% |
| QQYAAFPGA | 28.68 | 951.445 | 9 | 8.1 | 476.7365 | 47.71 | 6.35E+05 |  | 0.654068 | 0.02% |
| PQLQQVFIPPQ | 27.83 | 1293.7081 | 11 | -4.1 | 647.8625 | 101.04 | 6.26E+05 |  | 0.351397 | 0.02% |
| QIPRQLRC(+57.02)PAIHSM(+15.99)VHAIIM(+15.99)QQQQQQLVQAQQMGLVQ | 15.47 | 4366.2397 | 37 | 9 | 728.7248 | 108.14 | 6.21E+05 |  | 0.0408133 | 0.02% |
| AVRYFIGR | 28.11 | 980.5555 | 8 | 3.3 | 491.2896 | 27.97 | 6.17E+05 |  | 0.735335 | 0.02% |
| DWYKGPTLL | 26.17 | 1091.5651 | 9 | 6.4 | 546.7965 | 83.6 | 6.00E+05 | A0A411EWN3 | 0.708194 | 0.02% |
| QPFMQPLL | 23.02 | 972.5103 | 8 | 1 | 487.2657 | 83.4 | 5.78E+05 |  | 0.927075 | 0.02% |
| PREPQRDF | 21.21 | 1043.5148 | 8 | 0.7 | 522.7681 | 25.38 | 5.77E+05 | A0A1B2LQE8:A0A1B2LQC6:A0A1B2LQD6 | 0.529724 | 0.02% |
| GHVVNPI | 30.08 | 734.4075 | 7 | -4.6 | 368.2115 | 22.74 | 5.73E+05 |  | 0.196966 | 0.02% |
| Q(+42.01)GTGMVITGWAP | 18.48 | 1258.6016 | 12 | -1.6 | 630.3108 | 39.86 | 5.69E+05 |  | 0.359632 | 0.02% |
| VAPATQPRA | 16.59 | 909.5032 | 9 | -3.1 | 455.7601 | 20.42 | 5.52E+05 |  | 0.381001 | 0.02% |
| A(+42.01)QVQQQVFQPQ | 15.23 | 1341.6677 | 11 | 3.6 | 671.8475 | 100.42 | 5.14E+05 |  | 0.135928 | 0.02% |
| Q(+42.01)APVPVPAPHHPPPQ | 20.66 | 1609.8364 | 15 | 9.6 | 805.938 | 93.69 | 5.02E+05 |  | 0.613374 | 0.02% |
| QLLQPQLLQPQ | 20.43 | 1304.7452 | 11 | 0.4 | 653.384 | 84.17 | 4.95E+05 |  | 0.197895 | 0.02% |
| QVTQGIFQPQLQQVTQGIFQPQ | 17.89 | 2512.3074 | 22 | 8.2 | 629.093 | 91.6 | 4.78E+05 |  | 0.118124 | 0.02% |
| KDEHQRVQRF | 21.8 | 1341.6902 | 10 | 2.7 | 671.8582 | 101.6 | 4.76E+05 |  | 0.260752 | 0.02% |
| QQC(+57.02)C(+57.02)RQLEQIP | 17.71 | 1458.6708 | 11 | 9.3 | 730.3538 | 104.8 | 4.44E+05 |  | 0.317703 | 0.02% |
| EAVNWGL | 19.41 | 787.3864 | 7 | 9.2 | 394.7064 | 14.62 | 4.43E+05 |  | 0.442694 | 0.02% |
| YPVQGHINPML | 19.54 | 1267.6383 | 11 | -4.6 | 634.8273 | 103.36 | 4.32E+05 |  | 0.696077 | 0.02% |
| QPLM(+15.99)NRDGPA | 16.22 | 1113.5237 | 10 | -1.7 | 557.7715 | 108.35 | 4.17E+05 |  | 0.392209 | 0.02% |
| RMYLLENTGDFVRSNELQEGDFIVIYS | 15.83 | 3207.5547 | 27 | -3.2 | 642.52 | 8.37 | 4.13E+05 |  | 0.0115451 | 0.02% |
| QQPPFVQQEQPF | 22.39 | 1471.7095 | 12 | 9.7 | 736.8735 | 74.69 | 4.11E+05 |  | 0.478046 | 0.02% |
| LNASNPEWRHIN | 25.94 | 1449.7113 | 12 | -2.6 | 725.8654 | 37.31 | 3.87E+05 |  | 0.421062 | 0.02% |
| AALAEYFMYRERHTLIIYDDLSKQ | 23.42 | 2945.4746 | 24 | -8.2 | 590.1008 | 8.86 | 3.83E+05 |  | 0.0254443 | 0.01% |
| QPIMQQQQLLLQ | 15.44 | 1466.7915 | 12 | -6.2 | 734.4028 | 91.63 | 3.82E+05 |  | 0.277433 | 0.01% |
| Q(+42.01)IGGLIDLPGC(+57.02)PREPQ | 18.87 | 1790.8984 | 16 | -1.8 | 896.4602 | 104.47 | 3.73E+05 | A0A1B2LQE8:A0A1B2LQC6:A0A1B2LQD6 | 0.593628 | 0.01% |
| QPFVQQQPFVQQQP | 22.56 | 1697.8525 | 14 | 2.3 | 849.9405 | 94.74 | 3.61E+05 |  | 0.249283 | 0.01% |
| QPFVQQQQMFLQPL | 16.35 | 1730.8813 | 14 | 7.8 | 866.4598 | 104.84 | 3.52E+05 |  | 0.60177 | 0.01% |
| PGAAPYPMPPPQP | 15.66 | 1318.6379 | 13 | -5.8 | 660.3263 | 94.67 | 3.37E+05 |  | 0.717765 | 0.01% |
| QPQLQQEVFQPQ | 26.82 | 1468.731 | 12 | -4.2 | 735.374 | 101.61 | 3.37E+05 |  | 0.179808 | 0.01% |
| FQPQLQQVFQPQ | 15.37 | 1486.7568 | 12 | 2 | 744.3916 | 99.62 | 3.37E+05 |  | 0.312188 | 0.01% |
| QPQVQQQLIQPQ | 30.49 | 1433.7627 | 12 | -8.9 | 717.8865 | 106.47 | 3.29E+05 |  | 0.155929 | 0.01% |
| QPQVQQQLLQPQ | 30.49 | 1433.7627 | 12 | -8.9 | 717.8865 | 106.47 | 3.29E+05 |  | 0.197397 | 0.01% |
| GPVALASKDM(+15.99)AGRGSTSA | 15.48 | 1690.8308 | 18 | 2.8 | 564.6224 | 12.57 | 3.28E+05 |  | 0.249419 | 0.01% |
| QPYPEQQPF | 17.92 | 1132.5189 | 9 | 1 | 567.2706 | 99.27 | 3.18E+05 |  | 0.619288 | 0.01% |
| P(+42.01)GAAPYPMPPPQ | 18.07 | 1263.5957 | 12 | -6.7 | 632.8046 | 69.33 | 3.14E+05 |  | 0.806965 | 0.01% |
| QPFMQPLLQQQ | 15.42 | 1356.686 | 11 | 3.9 | 679.3569 | 99.49 | 3.05E+05 |  | 0.400656 | 0.01% |
| QPQTQM(+15.99)AGQVFIQPQ | 19.68 | 1715.8301 | 15 | 1.7 | 858.9288 | 78.53 | 3.05E+05 |  | 0.198575 | 0.01% |
| IMYLVAVIAMI | 17.75 | 1235.7021 | 11 | -8.9 | 618.8565 | 95.63 | 2.97E+05 |  | 0.234351 | 0.01% |
| L(+42.01)QAVC(+57.02)QVLIKVIFQ | 15.24 | 1699.9695 | 14 | 9.4 | 851.005 | 81.88 | 2.69E+05 |  | 0.341483 | 0.01% |
| G(+42.01)AAGPVNIAYSGVYQWWYTIGLRTN | 16.6 | 2798.3816 | 25 | 0.3 | 560.6871 | 12.52 | 2.69E+05 |  | 0.867911 | 0.01% |
| PPQPLAAAGFGVC(+57.02)PQ | 15.07 | 1508.7445 | 15 | 9.9 | 755.3915 | 110.58 | 2.64E+05 |  | 0.886238 | 0.01% |
| PQLEILAHGAT | 17.85 | 1148.6189 | 11 | 9.7 | 575.3257 | 108.47 | 2.57E+05 |  | 0.230454 | 0.01% |
| A(+42.01)GRARAAAAGFEKGIDRDL | 18.17 | 1986.0394 | 19 | 5.5 | 398.2197 | 11.54 | 2.45E+05 |  | 0.393019 | 0.01% |
| QPPFVQQEQPFVQQQP | 17.01 | 1923.9479 | 16 | -0.5 | 962.9865 | 95.04 | 2.42E+05 |  | 0.208715 | 0.01% |
| LWVAEDGMKM(+15.99)QGY | 19.11 | 1542.6847 | 13 | 0 | 772.3542 | 7.51 | 2.20E+05 |  | 0.38357 | 0.01% |
| QQPPFVQQEQPFVQQQQPFVQQQP | 21.12 | 2907.4304 | 24 | -2.4 | 970.1542 | 99.8 | 2.09E+05 |  | 0.203541 | 0.01% |
| QPQLQQQVFQLQLQQQ | 16.15 | 1981.0381 | 16 | 2.3 | 661.3588 | 92.32 | 1.97E+05 |  | 0.178691 | 0.01% |
| PQTQMAGQVFIQPQQLPQ | 18.17 | 2038.0305 | 18 | -0.7 | 1020.0279 | 102.49 | 1.95E+05 |  | 0.187326 | 0.01% |
| QPQLQQVFIPPQ | 21.49 | 1421.7666 | 12 | -8.8 | 711.8885 | 99.49 | 1.87E+05 |  | 0.373057 | 0.01% |
| LGRDSDTVAFVQFPQRFE | 16.99 | 2111.0435 | 18 | -9.1 | 1056.5256 | 103.83 | 1.78E+05 |  | 0.239377 | 0.01% |
| P(+42.01)RLVMHC(+57.02)P | 17.91 | 1050.5103 | 8 | 3.3 | 526.2673 | 8.23 | 1.77E+05 |  | 0.562154 | 0.01% |
| QQPLLQILQQ | 17.28 | 1207.6925 | 10 | -6.1 | 604.8534 | 94.71 | 1.71E+05 |  | 0.206787 | 0.01% |
| QPLAAAGFGVC(+57.02)PQ | 29.61 | 1314.639 | 13 | -6.5 | 658.3264 | 95.31 | 1.66E+05 |  | 0.747543 | 0.01% |
| QPFVQQQQMFLQPLLQQQLNPC(+57.02)KQFLVQQC(+57.02)SPVAAVPF | 15.16 | 4512.291 | 38 | 7.1 | 753.0656 | 109.26 | 1.63E+05 |  | 0.343693 | 0.01% |
| PQLQQQM(+15.99)LQTQLQQQMLQAQLQQQLLQAQLQQQLLQ | 16.73 | 4328.2734 | 36 | -2.8 | 722.3884 | 110.2 | 1.62E+05 |  | 0.109808 | 0.01% |
| QPQLQQVFQPQ | 19.48 | 1339.6885 | 11 | 3.4 | 670.8578 | 73.45 | 1.60E+05 |  | 0.226415 | 0.01% |
| QPQTQMAGQVFIQP | 22.51 | 1571.7766 | 14 | 8.2 | 786.9067 | 104.39 | 1.59E+05 |  | 0.24267 | 0.01% |
| QPFMQQQQMFLQPLLQQQLNPC(+57.02)KQFL | 16.51 | 3260.6296 | 26 | 6.9 | 816.1751 | 104.66 | 1.51E+05 |  | 0.445068 | 0.01% |
| PFVQQQPPFVQQEQP | 16.44 | 1795.8893 | 15 | -0.4 | 898.9569 | 97.18 | 1.49E+05 |  | 0.223204 | 0.01% |
| TAAQYGIEKEVDKGLKAAM(+15.99)EAGIDRKDLFVTSKIWRT | 18.18 | 4154.1831 | 37 | -8.8 | 693.3691 | 111.82 | 1.49E+05 |  | 0.0241229 | 0.01% |
| L(+42.01)GQGRDPSPGAVHLAEISDGYDR | 16.45 | 2451.1777 | 23 | -5.2 | 613.8021 | 11.1 | 1.43E+05 |  | 0.642741 | 0.01% |
| PGAAPYPM(+15.99)PPPQP | 16.51 | 1334.6328 | 13 | -2.6 | 668.3259 | 9.47 | 1.31E+05 |  | 0.717765 | 0.01% |
| PGAAPYPTPPPQPLAAAGFGVC(+57.02)PQP | 15.32 | 2457.2151 | 25 | 9.7 | 820.0918 | 109.67 | 1.26E+05 |  | 0.29315 | 0.00% |
| QPPFVQQEQPF | 23.19 | 1343.651 | 11 | 2.8 | 672.8386 | 110.5 | 1.22E+05 |  | 0.459777 | 0.00% |
| KDLLDNRKQRI | 18.7 | 1397.8103 | 11 | 9.6 | 466.9513 | 50.29 | 1.20E+05 |  | 0.256866 | 0.00% |
| QPIMQQQQLLLQQQQQM(+15.99)LQQQP | 19.42 | 2720.3738 | 22 | -1.8 | 907.8023 | 93.87 | 1.18E+05 |  | 0.136148 | 0.00% |
| GRASASAKKGSLRLDDWVLC(+57.02)RLYNKKNEWEKMQQGQQGEQKVEPKAE | 22.83 | 5473.7734 | 47 | 2.5 | 1369.4622 | 95.54 | 1.11E+05 |  | 0.621341 | 0.00% |
| QQQQQQQFIQP | 15.76 | 1399.6844 | 11 | -1.3 | 700.8527 | 97.36 | 1.10E+05 |  | 0.248523 | 0.00% |
| QQPPFVQQEQPFVQQQQQP | 25.41 | 2308.1235 | 19 | 6.4 | 1155.0833 | 100.16 | 1.05E+05 |  | 0.180447 | 0.00% |
| SREEILGRNC(+57.02)RFLQ | 15.84 | 1776.9053 | 14 | 2 | 889.4669 | 106.14 | 1.02E+05 |  | 0.533912 | 0.00% |
| A(+42.01)ALYRPA | 15.8 | 802.4337 | 7 | 4.2 | 402.2282 | 97.03 | 9.67E+04 |  | 0.494196 | 0.00% |
| PQLQQQLLQPQ | 17.23 | 1319.7197 | 11 | -8.5 | 660.8654 | 109.18 | 9.67E+04 |  | 0.182427 | 0.00% |
| PQLQQEVFQP | 16.02 | 1212.6139 | 10 | -3.9 | 607.3154 | 104.94 | 9.39E+04 |  | 0.196927 | 0.00% |
| KDLSLC(+57.02)HSMI | 17.08 | 1202.5787 | 10 | 4.2 | 602.3027 | 109.57 | 9.05E+04 |  | 0.476071 | 0.00% |
| GPVSC(+57.02)LSNTTHLL | 17.78 | 1397.6973 | 13 | 0.6 | 699.8605 | 107.88 | 9.03E+04 |  | 0.579789 | 0.00% |
| QIQNAMIHPQ | 21.34 | 1178.5867 | 10 | -5.8 | 590.3007 | 96.46 | 9.01E+04 |  | 0.244342 | 0.00% |
| QPAPAMLSSAPQ | 15.08 | 1196.5859 | 12 | 4.4 | 599.3064 | 94.54 | 8.91E+04 |  | 0.412106 | 0.00% |
| Q(+42.01)QVVQAQQMGLVQP | 16.81 | 1594.8137 | 14 | -2.1 | 798.4172 | 60.09 | 8.83E+04 |  | 0.129356 | 0.00% |
| Q(+42.01)KAHTQQGQWEQTQPQTSSSSSSFMIREVPPA | 16.67 | 3627.7012 | 32 | -7.2 | 605.6234 | 10.96 | 8.81E+04 |  | 0.0506349 | 0.00% |
| QPQLQQQAFQPQLQ | 18.1 | 1680.8584 | 14 | -2.7 | 841.4392 | 102.73 | 8.65E+04 |  | 0.311574 | 0.00% |
| KHKSLSAEIDRIKKENDNMQIELRHLKGEDVNSLQP | 16.78 | 4212.207 | 36 | 1.3 | 703.0469 | 106.86 | 8.65E+04 |  | 0.0337354 | 0.00% |
| VLFNNGTTDNTVRNFASSAS | 15.91 | 2114.0029 | 20 | 7.3 | 529.515 | 7.3 | 8.36E+04 |  | 0.0816959 | 0.00% |
| QKPHAVC(+57.02)VPFP | 16.61 | 1278.6543 | 11 | -6.2 | 640.3342 | 111.54 | 8.30E+04 |  | 0.703753 | 0.00% |
| QQQLQQQLIQPQ | 16.05 | 1478.7842 | 12 | -2.5 | 740.4019 | 96.94 | 8.05E+04 |  | 0.120573 | 0.00% |
| QPQTQMASQVFIQPQQLPQ | 20.62 | 2196.0996 | 19 | -6.6 | 733.04 | 96.7 | 8.01E+04 |  | 0.13612 | 0.00% |
| MLQAQLQQQLLQPQ | 15.91 | 1665.8872 | 14 | -6.5 | 833.9504 | 85.4 | 7.85E+04 |  | 0.133501 | 0.00% |
| TAAHIEK | 19.02 | 768.413 | 7 | 5.2 | 385.218 | 10.35 | 7.53E+04 |  | 0.076003 | 0.00% |
| LPQM(+15.99)SILAHAAVGGFLTHC(+57.02)GRNSLIEGLLFGHPLVM(+15.99)LPI | 17.29 | 4211.2207 | 39 | 4.3 | 702.8846 | 108.74 | 7.41E+04 |  | 0.0235659 | 0.00% |
| SREFTPSAADRIAGPISC(+57.02)LSNATHFLYFAAGY | 18.94 | 3489.6775 | 32 | 6.5 | 698.9515 | 7.99 | 7.08E+04 |  | 0.611347 | 0.00% |
| QPFVQQQQQQQPF | 18.86 | 1629.7899 | 13 | -7.1 | 815.9012 | 111.04 | 6.95E+04 |  | 0.322183 | 0.00% |
| TAKSGVGVASMG | 15.74 | 1063.5332 | 12 | -8.9 | 532.7723 | 8.15 | 6.82E+04 |  | 0.152179 | 0.00% |
| QPILQQQMLLQQQQQMLLQQQP | 15.14 | 2689.4043 | 22 | 2.1 | 1345.7202 | 92.13 | 6.62E+04 |  | 0.121086 | 0.00% |
| VAFPGQGHINPA | 15.82 | 1206.6145 | 12 | 4.8 | 403.2164 | 12.62 | 6.39E+04 |  | 0.370423 | 0.00% |
| EFPHPLARMPQ | 17.69 | 1321.66 | 11 | 1.8 | 661.8424 | 91.18 | 6.27E+04 |  | 0.614462 | 0.00% |
| Q(+42.01)GLGLQLPFST | 16.17 | 1201.6343 | 11 | 9.2 | 601.8335 | 75.58 | 5.81E+04 |  | 0.471138 | 0.00% |
| Q(+42.01)QFEC(+57.02)KIL | 15.3 | 1106.543 | 8 | 5 | 554.2848 | 80.08 | 5.39E+04 |  | 0.493214 | 0.00% |
| RSVGVPRLFFHVASC(+57.02) | 16.58 | 1730.9038 | 15 | 2.2 | 866.4662 | 106.97 | 5.30E+04 |  | 0.453019 | 0.00% |
| DKGLVVKSW | 15.56 | 1030.5811 | 9 | 6.1 | 516.304 | 81.26 | 5.05E+04 |  | 0.21077 | 0.00% |
| DKPIYVGFGSLP | 22.29 | 1291.6812 | 12 | 4.9 | 646.8549 | 108.44 | 4.76E+04 |  | 0.683467 | 0.00% |
| QQPIMQQQQLLLQQQQQMLQQQP | 18.41 | 2832.4375 | 23 | 0.3 | 945.1589 | 89.4 | 4.68E+04 |  | 0.149086 | 0.00% |
| QPLMILSASNC(+57.02)SQIGLFKNLKHPNAIKELNPRIPIQE | 20.32 | 4211.271 | 37 | -9.7 | 702.8831 | 110.5 | 4.65E+04 |  | 0.250518 | 0.00% |
| QPILQQQMLLQ | 19.56 | 1338.7329 | 11 | 3.8 | 670.3802 | 105.23 | 4.56E+04 |  | 0.339463 | 0.00% |
| MQIAHLEFRLVHPAPG | 19.29 | 1814.9613 | 16 | -6.3 | 908.4875 | 105.27 | 4.50E+04 |  | 0.418945 | 0.00% |
| QQPFVQQQQQQQP | 15.61 | 1610.7802 | 13 | -2 | 806.4005 | 95.57 | 3.98E+04 |  | 0.15412 | 0.00% |
| QPQAQM(+15.99)AGQVFIQP | 21.96 | 1557.761 | 14 | 8.9 | 779.8993 | 93.5 | 3.96E+04 |  | 0.396013 | 0.00% |
| SAPPPAHPQQQDAKNAAAKPPQ | 16.97 | 2248.1348 | 22 | -6.8 | 750.3849 | 97.19 | 3.78E+04 |  | 0.592289 | 0.00% |
| RSGSTEQQTTQDR | 18.59 | 1492.6866 | 13 | 3.9 | 747.3579 | 10.51 | 3.65E+04 |  | 0.0480761 | 0.00% |
| QPFVQQQPFVQQQQM(+15.99)FLQP | 16.96 | 2361.1575 | 19 | 8.7 | 788.0713 | 109.3 | 3.44E+04 |  | 0.385158 | 0.00% |
| AATQPQARPGLP | 15.03 | 1205.6516 | 12 | -3.8 | 603.8344 | 110.42 | 3.44E+04 |  | 0.383434 | 0.00% |
| TM(+15.99)KYLLMGGASSSILVH | 24 | 1822.9321 | 17 | 7.1 | 365.5984 | 8.63 | 3.31E+04 |  | 0.375599 | 0.00% |
| S(+42.01)PKVTRWTPQ | 16.09 | 1240.6564 | 10 | -5.7 | 621.3356 | 79.95 | 3.25E+04 |  | 0.269115 | 0.00% |
| EEVRIQEKSAPFPMYKSGPTSSRSS | 15.2 | 2797.3704 | 25 | -0.4 | 933.4692 | 15.3 | 3.21E+04 |  | 0.0564265 | 0.00% |
| QPQLQQQM(+15.99)LQTQLQQQM(+15.99)LQAQLQQQLLQAQLQQQLLQP | 21.48 | 4569.3794 | 38 | 1.4 | 914.8898 | 106.96 | 2.87E+04 |  | 0.14254 | 0.00% |
| QPQAQMAGQVFIQP | 20.57 | 1541.766 | 14 | 2.1 | 771.8965 | 108.21 | 2.48E+04 |  | 0.396013 | 0.00% |
| VGPSLSLHQC(+57.02)GLPL | 15.81 | 1476.7759 | 14 | 8.7 | 739.406 | 94.39 | 2.44E+04 |  | 0.759153 | 0.00% |
| QPQAQMAGQVFIQPQ | 22.44 | 1669.8246 | 15 | -7.5 | 835.9182 | 107.03 | 2.22E+04 |  | 0.321288 | 0.00% |
| PQLQQQAFQPQLQQVFNQP | 17.82 | 2266.1494 | 19 | 4.2 | 756.3981 | 104.51 | 2.09E+04 |  | 0.271641 | 0.00% |
| QPLMILSASNC(+57.02)SRIGLF | 15.29 | 1905.9805 | 17 | -9.1 | 953.9944 | 107.02 | 2.02E+04 |  | 0.832633 | 0.00% |
| YILKLIH | 18.81 | 898.564 | 7 | 6.5 | 450.2949 | 8.02 | 1.85E+04 |  | 0.223871 | 0.00% |
| QQQQQQFIQPQ | 16.02 | 1399.6844 | 11 | -6.7 | 700.8489 | 94.5 | 1.78E+04 |  | 0.229461 | 0.00% |
| TTFVTSW | 17.75 | 840.4018 | 7 | -6.5 | 421.2079 | 7.63 | 1.74E+04 |  | 0.166804 | 0.00% |
| GPVSC(+57.02)LSNATHFL | 16.87 | 1401.671 | 13 | 2.4 | 701.8486 | 107.75 | 1.68E+04 |  | 0.653071 | 0.00% |
| QQPFM(+15.99)QQQQQP | 17.17 | 1402.6299 | 11 | -3.2 | 702.3242 | 76.71 | 1.64E+04 |  | 0.210201 | 0.00% |
| R(+42.01)HGPGVSREGLGLHISQKLVKTMSGTVQYLREAESSSFI | 16.11 | 4296.2437 | 39 | 5.2 | 1075.0801 | 112.51 | 1.61E+04 |  | 0.246906 | 0.00% |
| SPRC(+57.02)IPFPLRY | 21.18 | 1404.7335 | 11 | 2.2 | 703.3798 | 51.77 | 1.60E+04 |  | 0.946027 | 0.00% |
| P(+42.01)YGQIFRPDNFVFGQSGAGNNWAK | 19.55 | 2711.2881 | 24 | -6.3 | 904.7696 | 12.43 | 1.56E+04 |  | 0.3931 | 0.00% |
| PGAAPYPTPPPQP | 16.42 | 1288.6451 | 13 | -1 | 645.333 | 93.32 | 1.43E+04 |  | 0.600363 | 0.00% |
| V(+42.01)DPLDSTSTM(+15.99)LQP | 15.84 | 1460.6704 | 13 | 3.2 | 731.3491 | 92 | 1.40E+04 |  | 0.282397 | 0.00% |
| QALPAMC(+57.02)DVYVPPQ | 15.66 | 1587.7426 | 14 | -0.5 | 794.8829 | 93.93 | 1.33E+04 |  | 0.417113 | 0.00% |
| PLAAAGFGVC(+57.02)PQ | 16.64 | 1186.5804 | 12 | 9.8 | 594.3068 | 106.14 | 1.21E+04 |  | 0.663317 | 0.00% |
| YVPPQC(+57.02)PVATAPL | 17.37 | 1411.7169 | 13 | -5.2 | 706.8663 | 107.01 | 1.12E+04 |  | 0.601241 | 0.00% |
| RSPDFSYGGPL | 19.36 | 1194.5669 | 11 | -2.7 | 598.2927 | 110.62 | 4.32E+03 |  | 0.827088 | 0.00% |
| TLNFEEYIH | 15.62 | 1164.5452 | 9 | -7.9 | 583.2787 | 8.07 | 4.15E+03 |  | 0.105095 | 0.00% |
| PQLQQQVFQPQLQQVFNQP | 18.8 | 2294.1807 | 19 | 1.7 | 765.74 | 108.86 | 3.24E+03 |  | 0.235588 | 0.00% |
| QPLLQQQMIP | 18.68 | 1194.6431 | 10 | 9.3 | 598.3379 | 107.94 | 2.86E+03 |  | 0.329555 | 0.00% |
| FSSLYPGC(+57.02)IATT | 17.86 | 1315.6118 | 12 | -8.4 | 658.8115 | 106.9 | 2.69E+03 |  | 0.482687 | 0.00% |
| P(+42.01)LGLLPPP | 30.59 | 844.5058 | 8 | -6.4 | 423.26 | 40.8 | 0 |  | 0.777458 | 0.00% |
| TSGNLFHVA | 27.73 | 944.4716 | 9 | 6.5 | 473.2489 | 20.9 | 0 |  | 0.269777 | 0.00% |
| QLQQVFNQPQ | 25.77 | 1228.62 | 10 | 0.1 | 615.321 | 84.61 | 0 |  | 0.148722 | 0.00% |
| HIEIPYP | 22.85 | 867.449 | 7 | -2.7 | 434.7332 | 30.03 | 0 |  | 0.412893 | 0.00% |
| IQPQLQQVFIPPQ | 22.59 | 1534.8507 | 13 | -7.4 | 768.4315 | 110.7 | 0 |  | 0.337911 | 0.00% |
| VPVGGATLGR | 22.55 | 925.5345 | 10 | -7.2 | 309.5184 | 14.2 | 0 |  | 0.333644 | 0.00% |
| QPQLQQQVFQPQLQQVFNQP | 21.06 | 2422.2393 | 20 | -9.3 | 1212.1228 | 102.88 | 0 |  | 0.267138 | 0.00% |
| QPILQQQLLL | 20.05 | 1192.7179 | 10 | -3.4 | 597.3677 | 85.23 | 0 |  | 0.493507 | 0.00% |
| QTLTAKMQSIPGA | 19.99 | 1344.7072 | 13 | -8.8 | 673.3589 | 91.71 | 0 |  | 0.122297 | 0.00% |
| GTLGILAGLFHLSVRPPQ | 19.7 | 1875.073 | 18 | -7.4 | 938.5424 | 111.92 | 0 |  | 0.518417 | 0.00% |
| P(+42.01)GAAPYPMPPPQPLAAAGFGVC(+57.02)PQP | 18.78 | 2529.2185 | 25 | -9.9 | 1265.6115 | 61.68 | 0 |  | 0.278115 | 0.00% |
| YNLM(+15.99)EYKDC(+57.02)GLSARAWWNNHRMQRITSAS | 18.61 | 3573.6453 | 29 | 4.6 | 894.428 | 11.9 | 0 |  | 0.275406 | 0.00% |
| VSAYIPT | 18.22 | 749.3959 | 7 | 9.2 | 375.7109 | 17.09 | 0 |  | 0.318765 | 0.00% |
| C(+57.02)(+42.01)QVARQQC(+57.02)C(+57.02)RQLAQIPEQLRC(+57.02) | 18.16 | 2743.2888 | 21 | -9 | 915.4341 | 11.52 | 0 |  | 0.579618 | 0.00% |
| QPPFVQQEQPFVQQQQPFVQQQP | 18.02 | 2779.3718 | 23 | -3.3 | 927.467 | 94.72 | 0 |  | 0.15062 | 0.00% |
| TVPEFPHVQFP | 18 | 1296.6503 | 11 | -9.5 | 433.2225 | 96.79 | 0 |  | 0.354506 | 0.00% |
| Q(+42.01)LQQVFQPQ | 17.87 | 1156.5876 | 9 | -8 | 579.2999 | 62.51 | 0 |  | 0.171951 | 0.00% |
| QMLQAQLQQQLLQPQ | 17.62 | 1793.9458 | 15 | -9.3 | 598.9872 | 71.22 | 0 |  | 0.121985 | 0.00% |
| FELKSHPYMGRAQDEFVNDRRRVRKEYD | 17.58 | 3540.7432 | 28 | -5.8 | 709.156 | 29.46 | 0 |  | 0.390148 | 0.00% |
| YFAEAVEEQKGVMKRHGDKIDHD | 17.36 | 2701.2917 | 23 | -4.4 | 1351.6552 | 55.33 | 0 |  | 0.106384 | 0.00% |
| V(+42.01)QQEQPFVQQQQPFVQQQPFVQQQQP | 17.28 | 3207.5737 | 26 | -9.4 | 802.8979 | 34.76 | 0 |  | 0.0921426 | 0.00% |
| QPQLQQQVFQQQVFQ | 17.05 | 1872.9482 | 15 | -2.8 | 937.4843 | 99.91 | 0 |  | 0.212352 | 0.00% |
| SLPLTRKDLM(+15.99)IVNMGPQ | 16.98 | 1928.0223 | 17 | -9.9 | 643.6788 | 59.19 | 0 |  | 0.353044 | 0.00% |
| QPQLQQQVFQQQLQQGVFQP | 16.97 | 2396.2236 | 20 | -9.7 | 1199.1145 | 106.42 | 0 |  | 0.243295 | 0.00% |
| PQLQLQQQVFQPQLQLQ | 16.84 | 2063.1165 | 17 | -5.4 | 1032.566 | 112.46 | 0 |  | 0.236758 | 0.00% |
| KDGQTREH | 16.71 | 969.4628 | 8 | -4.6 | 485.7393 | 19.31 | 0 | A0A411EWN3 | 0.116784 | 0.00% |
| GISIPMEDIGTSRVWSM(+15.99)RYRFWPNNKSRM(+15.99)YLLE | 16.39 | 4063.9858 | 33 | 9.1 | 1017.019 | 35.54 | 0 |  | 0.103573 | 0.00% |
| QQMFLQPPLQ | 16.32 | 1228.6274 | 10 | -6.5 | 615.3206 | 93.22 | 0 |  | 0.523742 | 0.00% |
| L(+42.01)LAIAVLLLPLM(+15.99)NHGINLAMAWEDQDFFKHC(+57.02)PLSHC(+57.02)SRHGPE | 15.85 | 4908.4126 | 42 | -9.9 | 1228.1056 | 111.14 | 0 |  | 0.480826 | 0.00% |
| PFSPPAPVADM(+15.99)QAANMPWPQQYAAFPGA | 15.84 | 2972.3625 | 28 | 8.1 | 1487.2094 | 99.48 | 0 |  | 0.152338 | 0.00% |
| Q(+42.01)QQQQQFFQPQ | 15.63 | 1475.6793 | 11 | -6.3 | 738.8467 | 82.03 | 0 |  | 0.371991 | 0.00% |
| QPQMQQVTQGIFQP | 15.59 | 1628.7981 | 14 | 7.6 | 815.4173 | 111.43 | 0 |  | 0.192113 | 0.00% |
| PQTSSSSSSFMIREVPPATNIAGNQP | 15.53 | 2702.2969 | 26 | -8.4 | 1352.1523 | 61.51 | 0 |  | 0.257956 | 0.00% |
| QPQLQQQVFQTQLQ | 15.52 | 1712.8845 | 14 | -7.6 | 857.4481 | 104.32 | 0 |  | 0.191295 | 0.00% |
| RC(+57.02)STMKDFPVTW | 15.44 | 1526.701 | 12 | -1.1 | 764.3615 | 90.32 | 0 | J7FQW2:Q9M4E2:J7FQW7:J7FK20 | 0.499382 | 0.00% |
| QPLAAAGFGVC(+57.02)PQP | 15.39 | 1411.6918 | 14 | 9.6 | 706.8641 | 109.79 | 0 |  | 0.749171 | 0.00% |
| SVGSRTVDELLLS | 15.38 | 1374.7355 | 13 | -3.2 | 688.3769 | 104.19 | 0 |  | 0.162817 | 0.00% |
| DEC(+57.02)RNIPVIM(+15.99)MSSQDSIGTVLKC(+57.02)M(+15.99)QNGAVDFLVKPVRKNEL | 15.34 | 4723.3262 | 41 | -3.9 | 1181.8412 | 23.35 | 0 |  | 0.21281 | 0.00% |
| QLQQVFILPQ | 15.14 | 1212.6866 | 10 | -3.8 | 607.3519 | 67.47 | 0 |  | 0.371638 | 0.00% |

Note: G (+42.01) indicates an acetylation modification on G resulting in an increase of 42.01 in the molecular weight of the peptide; M (+15.99) indicates an oxidative modification on M resulting in an increase of 15.99 in the molecular weight of the peptide; C(+57.02) indicates an carbamidomethylation modification on C resulting in an increase of 57.02 in the molecular weight of the peptide

Table S5. MOE Score, Number of formed hydrogen bonds, and Amino acid residues of peptides in OPs for docking with the Keap1 protein

| Peptide sequence | MOE docking score (kcal/mol) | Amino acid residues of the peptide | Number of formed hydrogen bonds | Amino acid residues of the Keap1 protein |
| --- | --- | --- | --- | --- |
| WGVGVRAERDA | -11.2253 kcal/mo | TRP1 | 5 | LEU557 |
|  |  | GLY2 |  | ILE559,LEU557 |
|  |  | ARG6 |  | VAL369,VAL608 |
| QPPFVQQEQP | -11.7040 kcal/mol | GLN1 | 7 | ILE416,LEU557,ALA510,VAL512 |
|  |  | GLN6 |  | VAL420 |
|  |  | GLU8 |  | ARG470 |
|  |  | GLN9 |  | HIS516 |
| QPQMQQQFFQPQ | -9.4300 kcal/mol | GLN5 | 10 | TRP497,ASN495 |
|  |  | GLN3 |  | MET499 |
|  |  | GLN7,GLN12 |  | PRO455 |
|  |  | GLN12 |  | SER439 |
|  |  | GLN12 |  | ASN438,HIS437 |
|  |  | PRO11 |  | ARG459 |
| QAGLYFL | -10.4001 kcal/mo | GLN1 | 8 | VAL604,VAL606,GLY367,VAL512,VAL465 |
|  |  | TYR5 |  | ARG326,GLY367 |
